# Meningeal cells and glia establish a permissive environment for axon regeneration after spinal cord injury in newts

**DOI:** 10.1186/1749-8104-6-1

**Published:** 2011-01-04

**Authors:** Katherine A Zukor, David T Kent, Shannon J Odelberg

**Affiliations:** 1 Interdepartmental Program in Neuroscience, University of Utah, Salt Lake City, UT 84132, USA; 2 Department of Internal Medicine, Division of Cardiology, University of Utah, Salt Lake City, UT 84132, USA; 3 Department of Neurobiology and Anatomy, University of Utah, Salt Lake City, UT 84132, USA

## Abstract

**Background:**

Newts have the remarkable ability to regenerate their spinal cords as adults. Their spinal cords regenerate with the regenerating tail after tail amputation, as well as after a gap-inducing spinal cord injury (SCI), such as a complete transection. While most studies on newt spinal cord regeneration have focused on events occurring after tail amputation, less attention has been given to events occurring after an SCI, a context that is more relevant to human SCI. Our goal was to use modern labeling and imaging techniques to observe axons regenerating across a complete transection injury and determine how cells and the extracellular matrix in the injury site might contribute to the regenerative process.

**Results:**

We identify stages of axon regeneration following a spinal cord transection and find that axon regrowth across the lesion appears to be enabled, in part, because meningeal cells and glia form a permissive environment for axon regeneration. Meningeal and endothelial cells regenerate into the lesion first and are associated with a loose extracellular matrix that allows axon growth cone migration. This matrix, paradoxically, consists of both permissive and inhibitory proteins. Axons grow into the injury site next and are closely associated with meningeal cells and glial processes extending from cell bodies surrounding the central canal. Later, ependymal tubes lined with glia extend into the lesion as well. Finally, the meningeal cells, axons, and glia move as a unit to close the gap in the spinal cord. After crossing the injury site, axons travel through white matter to reach synaptic targets, and though ascending axons regenerate, sensory axons do not appear to be among them. This entire regenerative process occurs even in the presence of an inflammatory response.

**Conclusions:**

These data reveal, in detail, the cellular and extracellular events that occur during newt spinal cord regeneration after a transection injury and uncover an important role for meningeal and glial cells in facilitating axon regeneration. Given that these cell types interact to form inhibitory barriers in mammals, identifying the mechanisms underlying their permissive behaviors in the newt will provide new insights for improving spinal cord regeneration in mammals.

## Background

Unlike mammals, adult newts have the remarkable ability to recover function after they are paralyzed by a spinal cord injury (SCI). After a complete transection injury, newts regenerate their spinal cords and regain use of their hindlimbs in as little as 4 weeks [1] (Additional file 1). This recovery requires supraspinal axons to regenerate across the lesion and re-establish connections with downstream targets and is not simply due to a reorganization of circuits within the spinal cord [1]. This finding led us to ask the question: why do axons regenerate across an injury site in the newt when they do not in mammals?

One of the main reasons why regeneration fails in mammals is because the environment of the injured spinal cord is inhibitory for axon regeneration [2]. After an SCI, a variety of cell types, including astrocytes and meningeal fibroblasts, react in ways that prevent axons from regenerating across the injury site. These reactive cells create physical barriers to regeneration, such as a glial scar and a glia limitans at the border between the cord and the injury site. They also create an extracellular matrix (ECM) that is inhibitory or repulsive for axon growth cone migration.

Therefore, axon regeneration may be enabled in the newt, in part, because the environment of the injury site is not inhibitory. Cells may respond in ways that help rather than hinder axon regeneration such that physical barriers are not created and the ECM is not inhibitory.

Much of what is known about spinal cord regeneration in salamanders comes from studies of tail regeneration. After tail amputation, a blastema forms and ependymoglia (EG) lining the central canal of the spinal cord elongate an ependymal tube that precedes and serves as scaffold for axon regeneration [3]. Regeneration in this context is thought to proceed as a recapitulation of developmental processes, and axons grow into newly developing tissues. Surprisingly, little is known about how axons regenerate after an SCI in the newt. In this context, axons must re-grow through an injury site having mature tissues on both sides of the lesion. This context is more relevant to the problem of spinal cord injury in humans. Older studies of SCI in the newt have noted that a blastema and glial scar do not appear to form [4], that axons can bridge large gaps in the cord before ependymal tubes elongate [5], and that, if left intact, the meninges can serve as a scaffold for axon regeneration [6]. A more recent study of SCI in the axolotl, a neotenic larval salamander, found that EG appear to undergo an epithelial to mesenchymal transition, migrate into the injury site to form a solid mass, and then undergo a mesenchymal to epithelial transition to re-form an ependymal tube that serves as a scaffold for axon regeneration [7,8]. In summary, previous studies suggest that physical barriers do not appear to form and EG and the meninges may help axons regenerate. Although O'Hara *et al*. [7] demonstrated that mesenchymal cells in the axolotl injury site were associated with fibronectin (FN), a permissive ECM protein, little else is known about the nature of the ECM of the injured newt spinal cord.

A focused and detailed study of axon regeneration and the cellular and extracellular environment axons encounter after an SCI in the newt has not been conducted. We, therefore, took advantage of modern labeling and imaging techniques to define, for the first time, stages of newt axon regeneration after a spinal cord transection injury and find that meningeal cells and glia, instead of interacting to form barriers to axon regeneration as they do in mammals [9,10], appear to interact to form a permissive environment for axon regeneration in the newt. Our study examines many aspects of newt spinal cord regeneration, establishes the required foundation for further investigation into the cellular and molecular mechanisms controlling this naturally occurring process, and may inform new therapeutic approaches in regenerative medicine.

## Results and discussion

To understand how newt axons regenerate after an SCI we carefully observed axons regenerating across a complete transection injury, which severed all innervation to the tail and hindlimbs. Animals were allowed to regenerate for 1 day, 3 days, 1 week, 2 weeks, 3 weeks, 4 weeks, 6 weeks or 9 weeks, and prior to tissue harvest an axon tracer was applied rostral or caudal to the original injury site in order to label descending or ascending regenerating axons, respectively. The tracer and nuclei were labeled fluorescently, and the injury site was analyzed in whole-mount preparation on a confocal microscope. At least three animals were observed per tracer application site and time point (Table 1).

**Table 1:** The number of animals observed at each stage and time point

Time point	Total	Retraction	Growth initiation	Wrapping	Wrapping/ wisping	Wisping	Wisping/ spiking (no ET)	Spiking (no ET)	Spiking (with ET)	Contact	Growth beyond injury (not recovered)	Growth beyond injury (recovered)
1 day	**6 (3/3)**	6 (3/3)										
3 day	**8 (4/4)**	6 (4/2)	2 (-/2)									
1 week	**15 (11/4)**	3 (3/-)	12 (8/4)									
2 week	**11 (6/5)**		2 (1/1)	3 (1/2)	2 (1/1)	4 (3/1)						
3 week	**10 (5/5)**			2 (2/-)		4 (-/4)			1 (1/-)	1 (1/-)	2 (1/1)	
4 week	**8 (4/4)**			4 (3/1)					1 (1/-)		3 (-/3)	
6 week	**7 (4/3)**					1 (-/1)	1 (-/1)	1 (-/1)	2 (2/-)	1 (1/-)		1 (1/-)
9 week	**6 (4/2)**										2 (1/1)	4 (3/1)
**Totals**	**71 (41/30)**	**15 (10/5)**	**16 (9/7)**	**9 (6/3)**	**2 (1/1)**	**9 (3/6)**	**1 (-/1)**	**1 (-/1)**	**4 (4/-)**	**2 (2/-)**	**7 (2/5)**	**5 (4/1)**

The numbers in parentheses break the totals for each category into rostral and caudal tracer application, respectively. For example, (4/2) indicates 4 animals received rostral tracer and 2 received caudal tracer. Animals that had recovered function were chosen preferentially for rostral tracer application. ET, ependymal tube.

### Spinal cord transection injury and axon tracer application

**Figure 1:**
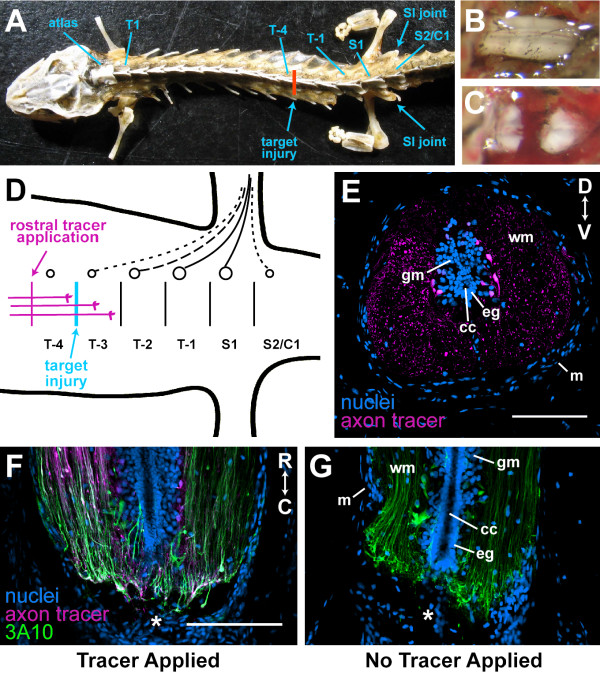
**Spinal cord transection injury and axon tracer application**. **(A) **Newt skeleton showing targeted location of injury. Note, the sacroiliac (SI) joints are not associated with the same vertebra in this animal, thus there are two sacral (S) vertebrae where there is usually one. T, trunk; C, caudal. **(B) **Intact and **(C) **completely transected spinal cord viewed from dorsal side. **(D) **Cartoon showing the location of the targeted injury, the spinal ganglia that supply innervation to the hindlimbs (solid line, primary contribution; dotted line, occasional contribution), and rostral tracer application site. **(E) **Cross-section of spinal cord about 500 μm away from a tracer application site showing the tracer labels axons and neurons specifically. **(F, G) **Longitudinal sections through 1-week regenerates. Asterisk, injury site. The tracer was applied to the animal in (F) and not to the animal in (G). Axons were also labeled with the 3A10 antibody. Application of the tracer 6 hours prior to tissue harvest did not alter the overall appearance of the axons at the injury site. cc, central canal; eg, ependymoglia; gm, grey matter; wm, white matter; m, meninges; D, dorsal; V, ventral; R, rostral; C, caudal. Scale bars: 200 μm ((F) and (G) are the same scale).

The spinal column in *Notophthalmus viridescens *contains one cervical (the atlas), about 13 trunk, 1 to 2 sacral, and 22 to 25 caudal vertebrae (Figure 1A) [11]. The numbers of trunk, sacral and caudal vertebrae appear to be variable. The hindlimbs are innervated primarily by the last two trunk (T-1 and T-2) and first sacral (S1) spinal nerves, though there can be contributions from the third to last trunk (T-3) vertebra and the vertebra after S1 (S2/C1) (Figure 1D) [11]. This is supported by the fact that the largest spinal nerves are associated with T-2, T-1 and S (Additional file 2) and axon tracer applied to the sciatic nerve labels primarily neurons in these spinal ganglia. To be sure all innervation to the hindlimbs was severed, we aimed to transect the spinal cord between T-4 and T-3 (Figure 1A, D), about 1 cm rostral to the hindlimbs. Upon dissection, it was often found that the injury was actually one or two segments rostral to the targeted site (Additional file 2B). Figure 1B shows what the intact spinal cord looks like when exposed from the dorsal side. After a complete transection injury, the two ends of the cut cord spring away from each other leaving a gap in the cord (Figure 1C). This type of injury was chosen because it is simple to perform consistently, spares no axons, severs the meninges as well as axons, and allows our studies to more seamlessly complement those done by previous investigators [1,4,12]. All injured animals were paralyzed and unable to swim for at least 3 weeks (Additional file 1), though the typical reflexive movements of the hindlimbs and tail were observed. Recovery of swimming function was scored and filmed in 6- and 9-week regenerates. One out of seven (14%) 6-week regenerates and four out of six (67%) 9-week regenerates recovered swimming function (Table 1). This recovery rate is consistent with that reported in Davis *et al*. [1].

**Figure 2:**
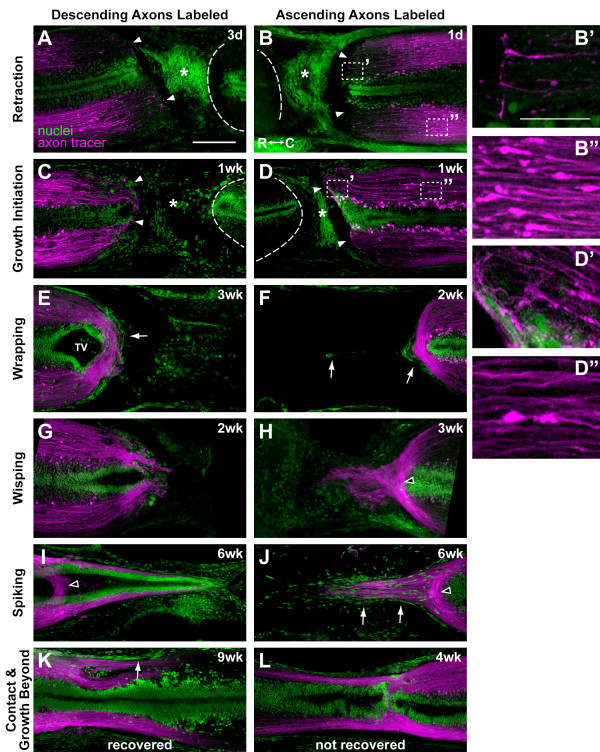
**Stages of axon regeneration**. Images are montages of single confocal planes (except where indicated) of longitudinal thick sections. Rostral is to the left. Descending (A, C, E, G, I, K) or ascending (B, D, F, H, J, L) axons were labeled with the axon tracer (magenta), and nuclei are shown in green. Time after injury is indicated in the upper right corner of each panel. **(A, B) **Retraction and **(C, D) **growth initiation stages. Arrowheads, end of cord; asterisk, injury site; dotted line, unlabeled cord opposite the injury site. (B) is a z-projection of four planes to highlight growth cones. **(B^'^, B^"^, D^'^, D^"^) **Enlargements of dotted boxes in (B) and (D) showing that growth cones (primed boxes) and dystrophic axons (double primed boxes) are present at both stages. **(E, F) **Wrapping stage. **(G, H) **Wisping stage. **(I, J) **Spiking stage. (H) and (I) are z-projections of two confocal planes to show wrapping axons with wisping (H) or spiking (I) axons. **(K, L) **Contact and growth beyond the injury site stage. The animal in (K) had recovered swimming function, while the one in (L) had not. Open arrowheads, residual wrapping axons; arrows, meninges. TV, terminal vesicle; R, rostral; C, caudal. Scale bars: 200 μm (A-L); 50 μm (B', B", D', D").

To label descending regenerating axons, a piece of gel foam saturated with the axon tracer biotinylated dextran amine (BDA) was inserted into a transection injury 1 segment (about 2 mm) rostral to the original injury site (Figure 1D), and the tracer was allowed to travel for 6 hours. Ascending axons were similarly labeled by applying the tracer one segment caudal to the original injury. BDA travels 3 to 4 mm in 6 hours (Figure 2K, L), produces a strong signal, and labels only neurons and their processes in the nervous system (Figure 1E). It does not diffuse across an injury site to label axons that have not regenerated across it (Figure 2A-D). Transecting the spinal cord to apply the tracer rostral or caudal to the original injury site 6 to 24 hours prior to tissue harvest does not appear to alter the general appearance of axons in the injury site (compare Figure 1F and 1G).

### Stages of axon regeneration

Our tracer analyses uncovered six stages of axon regeneration that appeared to be typical and sequential (Figure 2). The timing for all but the first two stages was highly variable (Table 1). This variability is likely due to differences in the age, size, health and activity level of the animals as well as the size of the gap in the cord and not due to differences in the completeness of the transection injury. It was quite easy to completely transect the newt spinal cord and visually confirm that the transection was complete (Figure 1C). The adult newts used in this study were collected from the wild and their ages are unknown. As noted below, the size of the gap in the cord tended to increase before it decreased, perhaps because of body movements of the animal. The times in parentheses after each stage indicate the earliest and latest time points at which the stage was observed.

#### Retraction stage (1 day to 1 week, most often before 1 week)

Axons appear to have retracted away from the end of the cut cord (Figure 2A, B), and many axon tips have a balled-up morphology that is reminiscent of the dystrophic end-bulbs seen on axons that are unable to regenerate (Figure 2B") [13,14]. Axon growth cones, however, can also be identified at this stage (Figure 2B').

#### Growth initiation stage (3 days to 2 weeks, most often at 1 week)

Axons appear to have initiated growth and grown back to the end of the cut cord (Figure 2C, D). As in the retraction stage, dystrophic axons and growth cones can be identified (Figure 2D', D"). The main difference between the first two stages is that many more axons extend to the end of the cut cord in the growth initiation stage. This difference is apparent even when all axons from the spinal cord are viewed in z-projections (Additional file 3). Also in this stage, the central canal, especially on the rostral side, begins to enlarge to form the terminal vesicle (TV).

#### Wrapping stage (2 weeks to 4 weeks)

Axons continue to grow and wrap around the end of the TV (Figure 2E, F). Residual numbers of dystrophic axons can still be seen at this and all subsequent stages. The meninges also wrap around, or bound, the end of the cord (Figure 2E, F) and often begin regenerating across the injury site ahead of the axons and TV (Figure 2F). The low density of cells within the injury site suggests that a scar is not forming. The size of the gap in the cord is also larger at this stage than at all other stages (Additional file 4). This could indicate that the wrapping stage is an abortive stage seen only in animals that do not recover function. We do not favor this interpretation, however, because wrapping axons are still evident at all subsequent stages, including the contact and growth beyond stage. This suggests that wrapping is a typical stage through which all regenerating spinal cords progress. The gap in the cord may get bigger before it gets smaller, simply as a result of body movements of the animal.

#### Wisping stage (2 weeks to 6 weeks)

Axons begin growing, or wisping, into the injury site ahead of the EG-lined TV (Figure 2G, H). Thus, as seen in previous studies of SCI in the newt, a pre-formed ependymal tube is not required for axon regeneration into the injury site [5,6]. Wisping axons, however, do not grow into the ECM of the injury site alone but rather remain associated with cells. Many of these cells appear to be continuous with the meninges. At this stage, the meninges do not bound the end of the cord as they did in the wrapping stage but continue regenerating across the injury site, preceding the regenerating axons. Some wisping axons even appear to follow the regenerating meninges (Additional file 5).

#### Spiking stage (3 weeks to 6 weeks)

As wisping axons continue to grow, they may fasciculate to form a spike (Figure 2I, J; Additional files 6 and 7). Spiking axons are still associated with cells in the injury site and cells that are continuous with the meninges. Some spikes do not contain an ependymal tube (Figure 2J), while others do (Figure 2I). In spikes with an ependymal tube, the ependymal tube appears to have elongated from the end of the cut cord because the largest part of the TV and residual wrapping axons are seen well behind the tip of the spike (Figure 2I; Additional file 7). It is conceivable that a spike may not need to form. For example, if the gap in the cord is not large, wisping axons may be able to grow through the lesion and enter the cord on the opposite side without forming a spike.

#### Contact and growth beyond the injury site stage (3 weeks to 9 weeks)

In this stage, the two ends of the cord make contact with each other, and axons begin growing through the cord on the opposite side of the injury to re-establish functional connections (Figure 2K, L). All animals that had recovered swimming ability were in this stage, although not all animals in this stage had recovered function. Also, in all animals that had recovered, the ependymal tubes on each side of the injury had fused together to re-establish a continuous central canal. It was not possible to correlate the distance axons had regenerated beyond the injury site with functional recovery in this study because the axon tracer appears to fade out after 3 to 4 mm (Figure 2K, L). This is true even in the intact spinal cord (data not shown). There is some tolerance for sloppiness in how axons regenerate, for even in animals that had recovered function, wrapping axons were still evident (Additional files 8A and 9), some axons appeared to decussate (Additional files 8A' and 9), and separate fiber tracts could be seen traveling along the meninges (Figure 2K). It would be interesting to determine whether these aberrant axon trajectories are pruned or refined over time in animals that have been allowed to regenerate for longer than 9 weeks. Excessive sloppiness or gross misalignments may be the cause of a delay in or a complete failure of functional recovery (Additional files 8B-F and 10).

### Sensory axons do not appear to regenerate

**Figure 3:**
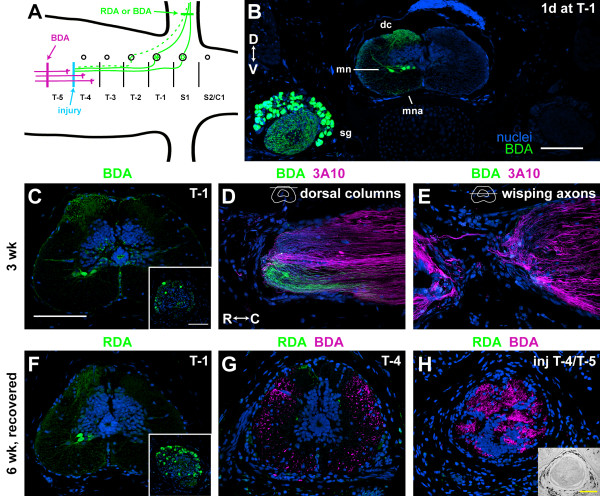
**Sensory axons may not regenerate**. (**A**) Cartoon showing tracer application sites in relationship to the SCI. Tracer was applied to the sciatic nerve in the hindlimb to label sensory axons (green) and, in 6-week regenerates, rostral to the injury to label descending axons (magenta). Tracer applied to the sciatic nerve labels primarily neurons in spinal ganglia S1 and T-1 (solid lines) and occasionally neurons in T-2 (dotted lines). **(B) **Cross-section through spinal cord and spinal ganglia at T-1, 1 day after SCI and tracer application to the sciatic nerve. The tracer labels sensory neurons in the spinal ganglia (sg), motor neurons (mn), dorsal column axons (dc), and motor neuron axons (mna) exiting to form the ventral root. **(C-E) **Sections through a 3-week regenerate. Sensory axons were labeled with biotinylated dextran amine (BDA; green), and all axons were labeled with 3A10 (magenta in (D, E)). (C) Cross-section at T-1. Inset, spinal ganglia on labeled side. (D, E) Longitudinal sections through the SCI at the level of the dorsal columns (D), and a more ventral level where there are wisping axons (E). Schematic cross-sections of the spinal cord in the upper right corner show where the section is along the D-V axis. **(F-H) **Cross-sections through a 6-week regenerate that had recovered swimming function at T-1 (F), at T-4, about 500 μm caudal to the SCI (G), and through the center of the SCI at T-4/T-5 (H). Sensory axons were labeled with rhodamine dextran amine (RDA; green), and descending axons were labeled with BDA (magenta). Inset in (F), spinal ganglia on labeled side. Inset in (H), phase image of injury site. Nuclei are shown in blue. D, dorsal; V, ventral; R, rostral; C, caudal. All scale bars: 200 μm ((C-H) are the same scale; insets in (C, F) are the same scale).

Descending and ascending regenerating axons appear to progress through similar stages (Table 1; Additional file 11). This was surprising given that sensory axons are not thought to regenerate after an SCI in newts or zebrafish even though these animals recover function [6,15]. To determine if ascending regenerating axons are true sensory axons, rather than axons arising from neurons within the spinal cord, an axon tracer was applied to the sciatic nerve 1 or 4 days prior to tissue harvest (Figure 3A). In 6-week regenerates, descending axons were also labeled with a different tracer. Our goal was to identify tracer-labeled sensory axons wisping into and growing through and beyond the lesion.

Tracer application to the sciatic nerve effectively labels, on the ipsilateral side only, sensory neurons in the spinal ganglia, motor neurons, dorsal column axons, and occasionally axons in a ventral location, which are presumably motor neuron axons exiting the cord to form the ventral root (Figure 3B). Labeled sensory and motor neurons were found mostly at the level of S1 and T-1 and occasionally at T-2 (Figure 3A).

To determine if sensory axons are among axons wisping into the injury site, 3-week regenerates received tracer application to the sciatic nerve 4 days prior to tissue harvest (n = 3). The spinal cord caudal to the injury site (T-2 to S1) was analyzed in cross-section to determine the effectiveness of the tracer application. Sensory and motor neurons and dorsal column axons were labeled in all three animals (Figure 3C). The injury site was analyzed in longitudinal sections to best view wisping axons. In sections through the dorsal columns, tracer-labeled sensory axons could be seen all the way up to the end of the cut cord (n = 2 out of 3; Figure 3D). In ventral sections through the region of wisping axons, however, none of the wisping axons were labeled with the tracer (Figure 3E).

To determine if sensory axons grow through and beyond an injury site, 6-week regenerates received rhodamine dextran amine (RDA) application to the sciatic nerve 4 days prior to tissue harvest. BDA was also used to label descending axons. The spinal cord caudal to the lesion and the lesion itself were analyzed in cross-section (n = 4). Sensory and motor neurons and dorsal column axons were effectively labeled with RDA in the spinal cord caudal to the lesion in three out of the four animals (Figure 3F). Two of the animals in which RDA was effectively applied had recovered function, and BDA-labeled descending axons could be seen growing through and at least 500 μm caudal to the injury site (Figure 3G, H). Despite this, RDA labeling in dorsal column axons got progressively weaker towards the injury, and RDA-labeled dorsal column axons were never seen in the injury site in any animal (Figure 3G, H).

We were not able to detect tracer-labeled sensory axons wisping into the lesion and growing through or beyond the lesion. This could indicate that sensory axons are either delayed or not regenerating or that the tracer faded out before the injury site. The tracer appears to travel a sufficient distance in four days to label wisping axons because it was seen robustly in dorsal column axons all the way up to the injury site in 3-week regenerates (Figure 3D). If the axons had regenerated, however, it is possible that the tracer was not detected in the injury site because of a dilution effect. The most likely explanation for why the tracer faded in the dorsal columns of the 6-week regenerates is because the dorsal column axons were degenerating [6]. Therefore, sensory information may not be needed for functional recovery in the newt, or sensory information may be transmitted to the brain via a relay circuit that is formed by ascending propriospinal axons that do regenerate across the injury site.

### Axons regenerate through white matter to reach functional targets

Axons regenerating beyond the injury site in zebrafish travel preferentially through the grey matter rather than white matter to reach functional targets [16]. Our initial study of axon regeneration in whole-mount preparations suggested that this is not true in newts (Figure 2K, L). To investigate this further, descending axons in three 6-week regenerates in the growth beyond the injury site stage were analyzed in cross-section about 500 μm caudal to the injury site. All axons in all three animals traveled in the white matter only (Figure 3G). Thus, axons do not re-route through the grey matter to reach functional targets in the newt. This suggests that white matter inhibitors [17] either are not present in the newt spinal cord during axon regeneration or that newt axon regrowth is not inhibited by these molecules.

### Extracellular matrix of the injured newt spinal cord

The ECM associated with an SCI in mammals is inhibitory or repulsive for axon growth cone migration [2]. Chondroitin sulfate proteoglycans (CSPGs) are a major component of this inhibitory ECM. After injury, they are expressed by a variety of cell types, including astrocytes and meningeal cells, and they are expressed in the glial scar and glia limitans that forms at the border between the injury site and the spinal cord. They are also upregulated in the spinal cord itself in a gradient that increases as axons approach the injury site [18,19]. ECM proteins that are canonically permissive for growth cone migration, such as FN [10,20], laminin (LM) [21] and collagen (Col) [22], as well as ECM proteins with more ambiguous effects, such as tenascin C (TN-C) [18], are also expressed. Overall, however, the ECM is dense and does not support growth cone motility [23,24].

Axon regeneration in the newt, then, may be enabled, in part, because the ECM of the injured newt spinal cord is not inhibitory. Consistent with previous studies, our initial axon regeneration experiments suggest that a dense scar does not form in the lesion (Figure 2E-J). To confirm this observation and determine if the ECM in the newt contains permissive rather than inhibitory proteins, we used immunohistofluorescence to analyze the expression of CSPGs, TN-C, FN, LM, and Col in wisping and spiking stage regenerates, the stages in which axons grow across the injury site. We used 2.5- to 3-week regenerates to target these stages (Additional file 11), and axons were labeled either with the axon tracer or an antibody against neurofilament associated protein (3A10).

**Figure 4:**
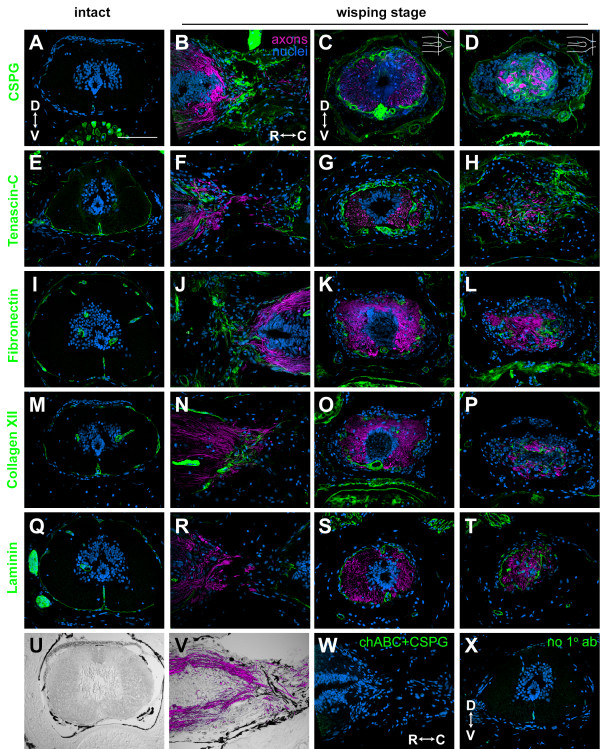
**Wisping axons are associated with loose ECM made up of canonically inhibitory and permissive proteins**. Axons were labeled with 3A10 (B-D, G-H, S-T, V) or the axon tracer (F, J-L, N-P, R) and are shown in magenta. Each ECM protein is shown in green, and nuclei are blue. **(A-D) **Chondroitin sulfate proteoglycan (CSPG) expression in the intact spinal cord (A, cross-section) and in wisping stage regenerates (B-D). (B) is a longitudinal section. (C) and (D) are cross-sections through the terminal vesicle (C), and axons wisping into the injury site (D) from the same animal. Schematic longitudinal sections of the spinal cord in the upper right corner of (C) and (D) show where the section is in relationship to the injury site. **(E-V) **Similarly, the expression of tenascin-C (E-H), FN (I-L), Collagen XII (M-P), laminin (Q-T), and pigment (U, V) is shown. **(W) **A section adjacent to the one shown in (B) treated with chondroitinase ABC (chABC) before incubation with the CS-56 antibody. **(X) **A section adjacent to the one shown in (E) treated only with secondary antibody; the primary antibody was omitted. D, dorsal; V, ventral; R, rostral; C, caudal. Scale bar: 200 μm (A-X).

In the intact spinal cord, CSPGs are not expressed, though they are expressed in the vertebral body in association with chondrocytes (Figure 4A). Surprisingly, CSPGs are expressed in the injured newt spinal cord. They are associated with the meninges and blood vessels (Figure 4B, C) and are expressed near wisping axons (Figure 4B, D). CSPGs are not found in the grey or white matter of the injured spinal cord and do not form a barrier between the cord and injury site. Thus, astrocytes in the spinal cord do not appear to express CSPGs. CSPGs also do not form a dense scar within the lesion.

Unlike CSPGs, TN-C, FN, Col, and LM are expressed in the intact cord in association with the meninges and blood vessels (Figure 4E, I, M, Q). After injury all of these proteins are expressed in a pattern similar to CSPGs. They are associated with the meninges and blood vessels (Figure 4F, G, J, K, N, O, R, S) and are expressed near wisping axons (Figure 4F, H, J, L, N, P, R, T). These proteins are not found in the grey or white matter of the injured spinal cord, do not form a barrier between the cord and injury site, and do not form a dense scar within the injury site. We occasionally detected a weak TN-C signal within the white matter of the spinal cord, but this signal was not significantly above background noise (autofluorescence and non-specific staining). A previous study detected a stronger TN-C signal in the white matter of the intact and regenerating newt tail spinal cord [25]. We were unable to reproduce these results, perhaps because we were analyzing a different region of the spinal cord, a different regenerative context, or TN-C expression with different TN-C antibodies.

In summary, similar inhibitory and permissive ECM proteins are expressed in the injured newt and mammalian spinal cords, but the pattern of expression is different. In the newt, the ECM associated with wisping axons is loose and open, and a dense scar does not form. Also, ECM proteins are not present within the grey or white matter of the spinal cord, and the ECM does not form a permanent barrier, or glia limitans, between the cord and the injury site, though we do have preliminary evidence that such a barrier may exist transiently during the wrapping stage (Additional file 12). Overall, even though inhibitory CSPGs are expressed, the ECM of the injured newt spinal cord does appear to be more permissive for axon growth cone migration than that of the injured mammalian spinal cord.

### Meningeal and endothelial cells are associated with wisping axons

**Figure 5:**
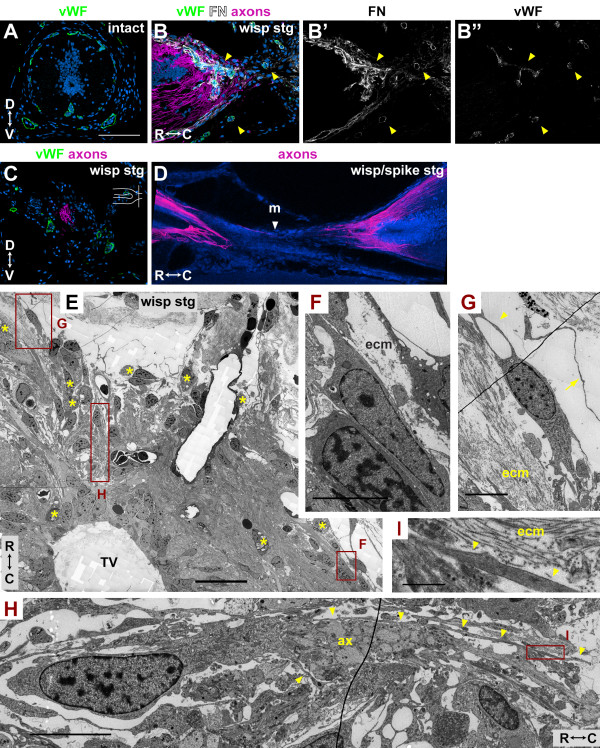
**Meningeal and endothelial cells are associated with wisping axons**. **(A-D) **Axons were labeled with the axon tracer (B, C) or 3A10 (D) and are shown in magenta, von Willeband factor (vWF) is green (A-C) and nuclei are blue. (A) Cross-section through intact spinal cord. (B) Longitudinal section through the SCI of a wisping stage regenerate labeled with anti-FN (white, shown separately in (B')) and anti-vWF (green, shown separately in (B")). Arrowheads, cells double-labeled with FN and vWF. (C) Cross-section through axons wisping into the injury site of a wisping stage regenerate. Schematic longitudinal section of the spinal cord in the upper right corner shows where the section is in relationship to the injury site. (D) Single confocal plane of a longitudinal thick section of a wisping/spiking stage regenerate. Arrowhead, meninges (m). **(E-I) **Longitudinal section through a wisping stage regenerate imaged with electron microscopy. (E) Region containing axons wisping ahead of the TV. Asterisks, phagocytic cells. (F) Enlargement of meningeal cells in box F of (E). (G) Enlargement of meningeal-like cell in box G of (E) that is associated with dura mater ECM (ecm). Arrowhead, closed loop formed by meningeal-like cell processes; arrow, process resembling dura mater cell process. (H) Enlargement of meningeal-like cell in box H of (E), rotated 90°. This cell's processes (arrowheads) wrap around a bundle of axons (ax) cut in cross-section. (I) Enlargement of box I in (H) showing that this cell's processes (arrowheads) are also associated with ECM (ecm). D, dorsal; V, ventral; R, rostral; C, caudal. Scale bars: 200 μm (A-D); 50 μm (E); 10 μm (F-H); 1 μm (I).

Given that cells closely associated with axons growing across the injury site and cells that create the permissive ECM are likely to be playing an important role in enabling axon regeneration, we sought to identify these cells. We hypothesized that they may be meningeal and/or endothelial cells because, in the intact and regenerating spinal cord, ECM appears to be expressed by cells of the meninges and blood vessels, not by cells in the grey or white matter (Figure 4). Thus, astrocytes and EG in the spinal cord do not appear to express ECM, at least not around their cell bodies. Our hypothesis is also supported by the fact that in the intact spinal cord, pigmented cells are only found in the meninges and blood vessels (Figure 4U), and pigmented cells are associated with wisping axons (Figure 4V). Additionally, many cells associated with wisping axons appear to be meningeal cells because they are continuous with the meninges (Figure 2). The meninges appear to regenerate ahead of regrowing axons, and some regrowing axons even appear to follow the regenerating meninges (Figure 5D; Additional file 5).

To determine if cells associated with wisping axons and ECM are endothelial cells, we used the endothelial cell marker von Willebrand Factor (vWF), which appears to specifically label blood vessels in the newt spinal cord (Figure 5A). In wisping stage regenerates, vWF+ cells can be found in the injury site and near (Figure 5B, B") and ahead of (not shown) regenerating axons. These cells are also associated with FN, though not all of the FN is associated with vWF+ cells (Figure 5B, B', B"). It has been hypothesized that blood vessels and nerves, because of their close proximity and parallel tracts, guide each other during development [26]. To determine whether they might guide one another during newt spinal cord regeneration, we analyzed injury sites in cross-sections. We found that though vWF^+ ^cells and axons are in the injury site at the same time, they do not need to be in direct contact with each other (Figure 5C). Therefore, regenerating blood vessels and axons do not appear to guide one another across the injury site, at least not through a contact-mediated mechanism.

Many cells in the injury site were not vWF^+^, and much of the FN was not associated with vWF^+ ^cells. We, therefore, sought to determine, more conclusively, if any of the cells closely associated with wisping axons and ECM were meningeal cells. Meningeal cell markers such as retinaldehyde dehydrogenase 2 (RALDH2) and NG2 [27] did not produce a signal in our preparations (Additional file 13) so we used electron microscopy (EM) to analyze cell types based on ultrastructure. In the intact and regenerating spinal cords, meningeal cells, in general, have an electron-dense cytoplasm filled with mitochondria, an elongated nucleus with some clumped chromatin, and thin dark processes that are often associated with ECM (Figure 5E, F; Additional file 14). None of the other cell types in the intact cord shared these characteristics of meningeal cells (compare Additional files 14 and 15). In regenerating spinal cords, we observed cells with meningeal cell characteristics closely associated with regrowing axons. Figure 5H shows an enlargement of one of these cells. It has an electron dense cytoplasm filled with mitochondria, an elongated nucleus with some clumped chromatin, and thin dark processes that appear to wrap around a bundle of axons that has been cut in cross-section. Processes from this cell are also associated with ECM (Figure 5I). Another meningeal-like cell (Figure 5G) was associated with dura mater-like elements, namely ECM and fine, dark cell processes (Additional file 14B). Processes from the cell in Figure 5G form a closed loop, which suggests they may be attempting to wrap or cup around structures in their environment.

It is difficult to know with certainty the identity of the meningeal-like cells. Stensaas [6] suggested that cells with these characteristics were dark astrocytes derived from EG. Morphologically, however, these cells and their processes resemble meningeal cells far more than they do EG and astrocytes (Additional files 15 and 16). Although these cells appear to wrap around axons, we and Stensaas [6] saw no evidence of them forming myelin, and therefore we do not think they are oligodendrocytes or Schwann cells. Further support for the idea that these cells may be meningeal cells comes from studies of meningeal cells that are transplanted into injured mammalian spinal cords along with olfactory ensheathing cells (OECs). Such meningeal cells appear to wrap axons into fascicles much like fibroblasts in the perineurium of peripheral nerves wrap axons [28,29]. These meningeal cells were also associated with ECM and had morphological features similar to our meningeal-like cells.

### Ependymoglial and astrocytic processes are associated with wisping axons

**Figure 6:**
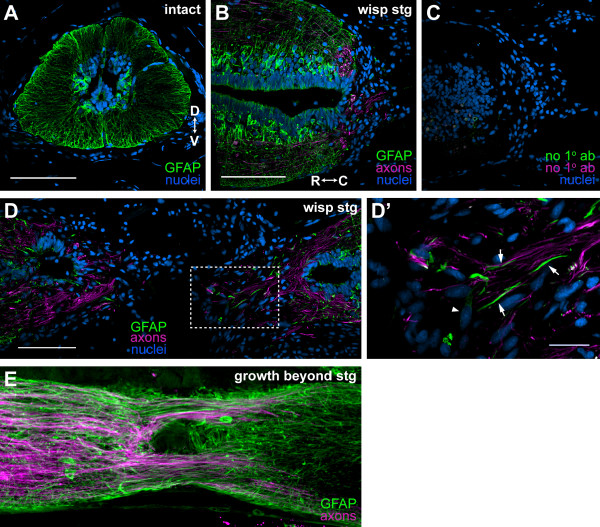
**Glial processes are associated with wisping axons and do not form inhibitory barriers**. Axons were labeled with 3A10 (B, D) or the axon tracer (E) and are shown in magenta. Glia were labeled with anti-GFAP (green), and nuclei are blue (A-D). **(A) **Cross-section through intact spinal cord. GFAP labels EG and astrocytes and all of their processes. **(B) **Longitudinal section through the SCI of a wisping stage regenerate. Astrocytes do not become hypertrophic (compare with (A)). **(C) **Section adjacent to (B) treated only with secondary antibodies; primary antibodies were omitted. **(D) **Longitudinal section through the SCI of a wisping stage regenerate. **(D') **Enlargement of dotted box in (D). GFAP^+ ^processes (arrows) are associated with wisping axons and do not form a glia limitans. Only a few weakly GFAP^+ ^cells (arrowhead) are in the injury site. **(E) **Single confocal plane of a longitudinal thick section of a growth beyond stage regenerate. GFAP^+ ^processes run parallel to axons across the injury site. D, dorsal; V, ventral; R, rostral; C, caudal. Scale bars: 200 μm (A-D; (B) and (C) are the same scale; (D) and (E) are the same scale); 50 μm (D').

As mentioned above, axons are often seen wisping ahead of the central canal, and thus, a pre-formed ependymal tube is not required for axon regeneration after an SCI. To determine if EG still might be playing a role in axon regrowth, we used an antibody against glial fibrillary acid protein (GFAP). In the intact newt spinal cord, GFAP weakly labels EG cell bodies, strongly labels astrocytic cell bodies and strongly labels radial processes from both these cell types (Figure 6A). Therefore, this cell marker enabled us to observe the astrocytic as well as EG response.

In the injured spinal cord, GFAP expression is maintained in astrocytes and EG around the central canal. It does not appear to be upregulated, and GFAP^+ ^processes do not appear to be thicker than they are in the intact cord (compare Figure 6A and 6B). Therefore, astrocytes do not appear to become hypertrophic. In the injury site, only a few GFAP^+ ^cell bodies can be found (Figure 6D, D'). These cells are more likely to be EG than astrocytes because they express GFAP only weakly. Thus, astrocytes do not migrate into the lesion. In wisping stage regenerates, GFAP^+ ^processes are closely associated with wisping axons (Figure 6D, D'). These processes likely originate from EG and astrocytes that surround the central canal. Rather than being aligned perpendicular to axons, as they are during tail regeneration, however, the GFAP^+ ^processes are aligned more parallel to the direction in which axons travel. This is especially evident in later-stage regenerates (Figure 6E). Note also that the GFAP^+ ^processes do not form a glia limitans between the cord and the injury site as they do in mammals (Figure 6B, D).

These data demonstrate that, while the EG response after SCI appears to be different to that after tail amputation, EG are still likely to be playing an important role in enabling axon regeneration because GFAP^+ ^processes are closely associated with axons growing across the injury site. Also, while EG do not appear to migrate into the injury site in large numbers, there are a few that appear to do so.

Furthermore, the astrocytic response in the newt is very much different to that seen in mammals. Newt astrocytes do not appear to become hypertrophic, migrate into the injury site, express ECM, or form a glia limitans or scar. Instead of inhibiting axon regeneration as they do in mammals, newt astrocytes may, like EG, play a supportive role because many of the GFAP^+ ^processes associated with axons likely arise from astrocytes as well as EG.

### An inflammatory response is present but does not appear to be detrimental to regeneration

It has been hypothesized that inflammation leads to scarring and non-regenerative wound healing and that it is not present in systems that regenerate [30]. In support of this, macrophages in the injured mammalian spinal cord contribute to the formation of the scar [31] and have even been shown to physically interact with axons to cause retraction [32].

**Figure 7:**
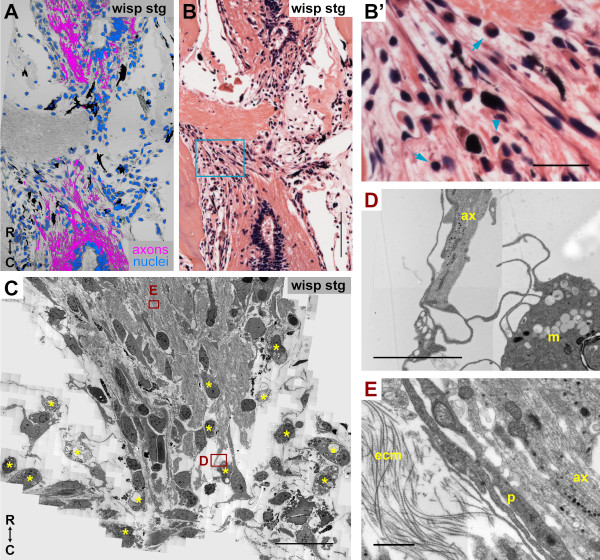
**An inflammatory response is present but not detrimental to axon regeneration**. **(A) **Longitudinal section through a wisping stage regenerate. Axons were labeled with 3A10 (magenta), nuclei are in blue, and fluorescent channels were laid over the phase image. **(B) **A section adjacent to (A) stained with H&E. Many small cells with dark blue nuclei are present near wisping axons. **(B') **Enlargement of blue box in (B). Lymphocytes (arrowhead) and monocytes (arrows) can be identified near wisping axons. **(C-E) **Longitudinal section through a wisping stage regenerate imaged with EM. (C) Region containing axons regenerating into the injury site. Many phagocytic cells (asterisks), or macrophages, can be identified near regenerating axons. (D) Enlargement of box D in (C). Processes from this macrophage (m) are in direct contact with regenerating axons (ax). (E) Enlargement of box E in (C). Another example of a meningeal cell-like process (p) that is mingled and closely associated with regenerating axons (ax). ecm, ECM; R, rostral; C, caudal. Scale bars: 200 μm (A, B); 50 μm (B',C); 5 μm (D); 1 μm (E).

To determine if an inflammatory response might be present in the newt SCI, we used H&E staining. With H&E staining, an inflammatory response is characterized by the appearance of many small round cells that have dark blue nuclei and very little cytoplasm. Lymphocytes have almost no cytoplasm, whereas monocytes have a small rim of cytoplasm. Based on these characteristics, an inflammatory response does appear to be present in wisping stage regenerates (n = 5; Figure 7A, B, B'). Figure 7B' highlights some of the more obvious examples of lymphocytes and monocytes that are present at this stage. These types of cells are associated with the fibrin clot that forms in the injury site and can be identified as early as one week after injury (Additional file 17A, B-B").

To confirm the presence of various types of inflammatory cells, we again tried several cell-type specific antibodies, but these did not produce a reliable signal in our preparations (Additional file 13). We were, however, able to identify many phagocytic cells, or functional macrophages, in the region of regenerating axons in our EM images. Asterisks in Figures 5E and 7C mark obvious macrophages. These macrophages could even be seen to be in direct contact with axons (Figure 7D). Figure 7E shows another example of a meningeal-like cell process that is closely associated with regenerating axons as well as ECM.

These data demonstrate that an inflammatory response does appear to be present in the injured newt spinal cord, but does not appear to be detrimental to the regenerative response. In fact, one of the most severe cases of inflammation was seen in a contact stage regenerate (Additional file 17C,C'). The inflammation, which was likely present at earlier stages as well, did not prevent regeneration from progressing to this advanced stage. The interaction of newt macrophages with axons, if it also causes axon retraction in newts, does not prevent the net response to be that of axon growth. Perhaps the beneficial effects the immune system can have on regeneration [33] outweigh any detrimental effects in the newt.

## Conclusions

**Figure 8:**
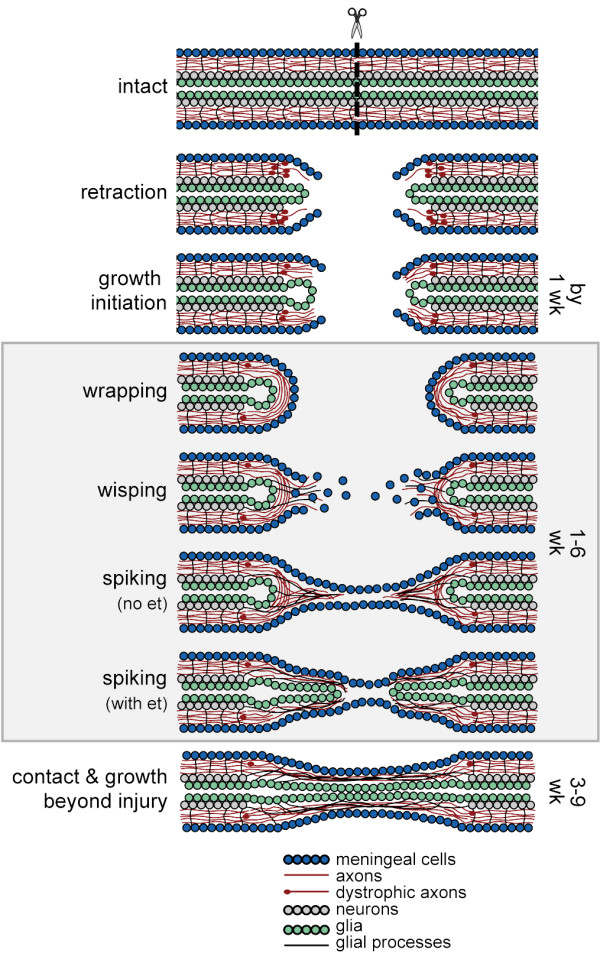
**Cellular model of newt axon regeneration**. Schematics of longitudinal sections through the spinal cord are shown. See text for details. et, ependymal tube.

Axons appear to be able to regenerate after an SCI in the newt in part because the environment of the injury site is not inhibitory. Instead of forming a dense inhibitory scar and interacting to form a glia limitans and as they do in mammals [2,9,10], newt meningeal cells and glia appear to create a permissive environment for axon regeneration. Figure 8 summarizes the stages of axon regeneration and the roles these cell types play. After an SCI, axons initially retract from the end of the cut cord and appear dystrophic, but then initiate growth by about 1 week. Axons wrap around the end of the TV before growing into the lesion, perhaps because meningeal cells create a transient basal lamina. Meningeal and endothelial cells lead the way across the lesion and are associated with a loose extracellular matrix that permits growth cone migration. Axons grow into the injury site next and are closely associated with meningeal cells and glial processes that extend from EG and astrocytic cell bodies surrounding the central canal. Later in the process, ependymal tubes lined with glia extend into the lesion as well. Then, as a unit, meningeal cells, axons, and glia close the gap in the spinal cord. Finally, axons enter the cord on the opposite side of the injury and travel through white matter to reach functional targets. We also noted that though ascending axons do regenerate, sensory axons do not appear to be among them and that, overall, this regenerative process can occur in the presence of an inflammatory response.

### Model

Based on our observations, we propose a glia-meningeal cell interaction model of spinal cord regeneration: spinal cord regeneration is enabled when meningeal cells and glia (astrocytes and EG) interact to form a conduit for axon regeneration rather than an inhibitory barrier (scar, glia limitans, and dense ECM). Simpson [34] hypothesized that successful spinal cord regeneration depends upon an interaction between mesenchymal cells and the ependymal epithelium that stimulates the ependyma to proliferate and form a scaffold, which guides axons across the lesion. Since astrocytes also appear to be an important component in this interaction, we have included astrocytes with the ependymal cells and refer to them as glia. Simpson postulated that blastemal cells are the important mesenchymal cell after tail amputation, but was not specific about which cells play that role after SCI. We suggest that meningeal cells are an important mesenchymal cell after SCI and that they, too, can provide a scaffold for axon growth and guidance.

### Glia

Our model of spinal cord regeneration after an SCI is similar to that after tail amputation in that EG play a central role. Their processes are closely associated with regenerating axons and may provide a substratum for growth cone migration and guidance via cell-cell adhesion. Our data also suggest that astrocytes may play a similar role. These astrocytes may be immediate descendants of EG and not the fully mature astrocytes seen in the mammalian spinal cord [35], and this may explain, in part, why they respond so differently to SCI. The arrangement of the glial processes, however, is different from the tail regeneration model, at least initially. Instead of extending radially from a pre-formed ependymal tube to form channels through which axons can grow [3], they extend longitudinally ahead of the TV and run roughly parallel to the regenerating axons. Such glial support may allow the axons to grow into the injury site in the absence of a pre-formed tube. They may also help guide the EG lining the central canal, and thus the ependymal tube, into the region of wisping axons, similar to the way in which radial processes guide newborn neurons to their proper locations in the developing cortex. Soma may translocate as their processes shorten, or they may migrate along the processes of other EG [36]. Given that very few cells in the injury site express GFAP, we do not think many EG undergo an epithelial to mesenchymal transition to migrate freely into the lesion as proposed for axolotls after SCI [7]. This does not rule out the possibility, however, that undifferentiated progeny of EG might migrate into the injury site. Alternatively, given that the mesenchymal cells in the axolotl injury site were also GFAP^- ^and were associated with FN, such cells could be meningeal and/or endothelial cells.

### Meningeal cells

Unlike most previous models of salamander spinal cord regeneration, our model assigns an important role to meningeal cells. Although they are thought to be detrimental to axon regeneration in the injured mammalian spinal cord, meningeal cells are able to stimulate, support, and guide developmental and regenerative processes in other contexts. Meningeal cells are fibroblast-like, and fibroblasts in flesh wounds are responsible for remodeling the ECM into either scar or normal tissue. The difference appears to depend on whether the fibroblasts are stimulated by transforming growth factor (TGF)β1 or TGFβ3 [37]. As mentioned previously, meningeal cells harvested from the olfactory bulb, a part of the nervous system that does regenerate, and transplanted into a mammalian SCI along with OECs appear to fasciculate axons into perineurial-like structures and enhance the ability of OECs to remyelinate axons [28,29]. During development, the meninges serve as substratum for and secrete a chemoattractant, CXCL12, which regulates the migration of Cajal-Retzius cells [38]. In the developing forebrain, the meninges secrete retinoic acid, which stimulates radial glia to begin producing neurons [39]. Meningeal cells in mammalian SCI express retinoic acid and permissive ECM proteins, such as LM and FN [27,40]. Retinoic acid has been shown to stimulate and possibly be an attractant for neurite outgrowth [41,42]. While the permissive ECM proteins expressed by meningeal cells *in vitro *tend to form a basal lamina that axons cannot cross, axons appear to grow quite well along it [40]. Thus, the basal lamina can either guide or inhibit axon regeneration depending on its orientation. In fact, following a large ablation of the spinal cord in which the meninges were left intact, axons were seen regenerating along the basal lamina of the meninges, which was oriented in a productive direction parallel to axon regrowth [6].

### Axons

Our model does not exclude the possibility that axons and axon intrinsic properties also play an important role in newt spinal cord regeneration. The 1 week required for growth initiation suggests that an intrinsic growth potential is activated. This is supported by the fact that a 1-week conditioning lesion encouraged neurite outgrowth from newt retinal explants [43]. Activation of intrinsic growth could also enable axons to overcome any inhibition CSPGs might inflict, similar to the way in which embryonic neurons adapt to CSPGs [44,45]. Furthermore, matrix metalloproteinases are upregulated during newt spinal cord regeneration (unpublished observations), and if they are expressed in growth cones, they could breakdown inhibitory ECM molecules and allow the axons to grow through regions that would normally be non-permissive. Axons may also directly influence the behavior of the cells in their environment. Nerves are required for newt limb regeneration, and they stimulate Schwann cells to produce anterior gradient protein, a factor that is sufficient to rescue regeneration in de-nervated limbs [46]. Regenerating optic nerve axons in the frog appear to stimulate a breakdown of the blood brain barrier at the front of regenerating axons [47]. Finally, when degenerated optic nerve fragments containing hypertrophic astrocytes are implanted into an optic nerve injury site in frogs, axons grow into the grafted tissue and appear to stimulate the reactive astrocytes to adopt a more permissive architecture: the astrocytes extend radial processes and form compartments for axon elongation [48]. Thus, newt axons may play a role in stimulating appropriate glial and meningeal cell responses.

If meningeal cells and glia support the development and regeneration of the nervous system in some contexts, why do they not do so in all contexts? Although no definitive answer can be put forward, we suspect that the state of differentiation of the cells may play a key role. In the newt spinal cord, EG maintain characteristics of radial glia of the developing cortex [49], astrocytes may be recent progeny of EG [35], and many differentiated cells have the ability to dedifferentiate and become more progenitor-like [50]. An exciting new study in mouse suggests that mammalian glia, and possibly mammalian meningeal cells, may retain the capacity to respond to an SCI in ways that support axon regeneration. When the tumor suppressor gene *PTEN *(phosphatase and tensin homolog) is deleted in mouse corticospinal neurons, the neurons are able to regenerate axons across an SCI, and their axons cross the injury site in close association with GFAP^+ ^cells and cell processes [51]. These GFAP^+ ^bridges tended to appear around the edges of the spinal cord, the regions that are closest to the meninges. Learning more about how newt cell types are intrinsically different from their mammalian counterparts and what factors stimulate newt cell types to respond and interact in ways that promote axon regeneration may help us develop methods to cultivate similar behaviors in mammalian cells. Identifying effective treatments for spinal cord injury in mammals has been so intractable that it is imperative we take full advantage of the opportunity to learn how nature has already solved this problem in newts.

## Materials and methods

### Animals

Adult newts, *N. viridescens*, were purchased from Charles D Sullivan Co. Inc. (Nashville, TN, USA), housed at 22°C in glass aquariums equipped with water filters, and fed live blackworms, *Lumbriculus varietgatus *(Eastern Aquatics, Lancaster, PA, USA). All animal protocols were approved by the University of Utah Institutional Animal Care and Use Committee.

### Spinal cord injury

Newts were anesthetized by submersion in 0.1% Tricaine (ethyl 3-aminobenzoate methanesulfonate salt; Sigma, St. Louis, MO, USA) in 20 mM Tris-HCl, pH 7.5 for 10 minutes and placed in ice for 30 minutes prior to surgery. Complete transection injuries were performed similar to those described in Davis *et al*. [1,12]. After swabbing the skin with 10% providone iodine, a deep incision was made between vertebral bones about 1 cm rostral to the hindlimbs. Using forceps, the vertebrae were gently and slightly separated and the dorsal lamina of the caudal vertebra was removed to expose the spinal cord. The spinal cord was cut completely with fine spring scissors. The completeness of the transection was easily verified by visual inspection (Figure 1C). Blood was irrigated with sterile 85% PBS when necessary. Animals were kept moist during the procedure, placed on ice for 15 minutes after the surgery, and allowed to recover in bins that were tilted to produce a shallow area of water. Bins were cleaned and filled with fresh de-chlorinated water every day or every other day throughout the recovery period.

### Axon tracer application

Our procedure was modified from that used in zebrafish [15,16]. Three axon tracers were tried: BDA (3 kDa, lysine fixable, Invitrogen, Carlsbad, CA, USA, D7135), RDA (3 kDa, lysine fixable, Invitrogen, D3308), and biocytin (Pierce, Rockford, IL, USA, #28022). RDA appeared to work as well as BDA, but was only used when two tracers were needed in the same animal. Biocytin, which was reported to reveal thin processes better than a 10 kDa RDA [15], unfortunately did not produce a signal in our preparations, perhaps because of the decalcification step in tissue processing (see below). BDA and RDA were prepared similarly: small pieces of gel foam (Pharmacia and Upjohn Co., Kalamazoo, MI, USA) were soaked in 1.5 μl of a 10% solution and allowed to air dry 40 minutes in a tissue culture hood to further concentrate the solution. Fast green dye (Fisher, Waltham, MA, USA, F-99) was added to the BDA solution at 0.05 mg/ml to make it visible *in vivo*. Biocytin did not readily go into solution at 10%, but a slurry was made. Small pieces of gel foam were soaked in 10 μl of the slurry, air dried completely, and stored at -20°C. To apply tracer to the spinal cord, the cord was transected one vertebral segment (about 2 mm) rostral or caudal to the original injury site using the same procedure described above and a piece of gel foam saturated with tracer was inserted into the gap. To apply tracer to the sciatic nerve, an incision was made along the course of the sciatic blood vessel on the posterior aspect of the hindlimb where the hindlimb joins with the body. Fine forceps were used to tease the nerve from the vessel, and the nerve was transected with fine spring scissors. A piece of gel foam saturated with tracer was applied and the wound was sealed closed with Vetbond tissue adhesive (3M, St. Paul, MN, USA). Excessive bleeding was controlled before tracer application, if necessary, by inserting a piece of gel foam into the wound and allowing the animal to sit on ice for several minutes. Use of this technique was minimized, however, because it decreased the efficiency of tracer uptake.

### Tissue harvest, fixation, and decalcification

Animals were anesthetized as above, placed on ice for at least 15 minutes, then perfused with about 1.5 ml 85% PBS followed by 3 ml periodate-lysine-paraformaldehyde (PFA) fixative (PLP: 75 mM lysine, 10 mM sodium periodate, 0.5% PFA in 85% PBS, pH 7.4; 0.1 M lysine and 2% PFA stocks were stored at -20°C and ingredients were mixed and used within 2 hours) [52]. All incubations were performed rocking at room temperature (RT) and rinses were for 20 to 30 minutes, three times, unless otherwise specified. The spinal column was harvested in about 4 mm long segments, post-fixed in PLP for 2 hours, rinsed with PBS (137 mM NaCl, 2.7 mM KCl, 8.1 mM Na_2_HPO_4_, 1.15 mM KH_2_PO_4_, pH 7.4), rinsed in fresh PBS overnight at 4°C, decalcified with Morse's solution (22.5% formic acid, 10% sodium citrate) for 24 hours, and rinsed with PBS. Some of the spinal cords prepared for paraffin sections were not perfusion fixed and this did not seem to affect the results, though perfusion did make tissue prepared for thick and frozen sections easier to section. An alternative fixative, 4% PFA, was tried, but the quality of sections was not improved, there was more autofluorescence, and some antibodies did not work as well.

### Thick sections and fluorescent labeling (spinal cord whole-mounts)

Methods were adapted from previous studies [53,54] and described in Zukor *et al*. [55]. All incubations were performed rocking at RT unless otherwise specified. Decalcified tissue was bleached with formamide bleach (6% hydrogen peroxide, 3% formamide, 0.1% Triton X-100 in PBS) for about 2 hours; rinsed with PBS for 20 to 30 minutes, three times; and embedded in 4% agarose. Longitudinal sections containing the whole spinal cord (about 350 μm thick) were cut on a vibratome and collected in ice cold PBS. Sections were transferred to 0.67 ml centrifuge tubes, permeabilized with PBS-TxD (PBS containing 0.5 to 2% Triton X-100 and 20% DMSO; 0.5% Triton X-100 was sufficient for streptavidin, 2% was necessary for antibodies) overnight and incubated in streptavidin or primary antibody diluted in PBS-TxDB (PBS-TxD containing 1% BSA (EMD, Gibbstown, NJ, USA, #2960) and 0.1% fish skin gelatin (Sigma, G7765) as blocking reagents) for 7 days for streptavidin or 10 days for antibody penetration. Sections needing incubation with secondary antibodies were rinsed with PBS-Tx (PBS with 2% Triton X-100) for 30 minutes, five times and incubated in secondary antibody diluted in PBS-TxDB for 10 days. Sections were then rinsed in PBS for 30 minutes, four times; rinsed in TNT (100 mM Tris-HCl, pH 7.5, 150 mM NaCl, 0.05% Tween-20) for 30 minutes; incubated in SYTOX green (Invitrogen, S7020) diluted 1/1,000 in DMSO, then 1/25 in TNT for 3 hours to stain nuclei; and rinsed in TN (TNT without Tween-20) for 10 minutes, three times. Sections were transferred to glass vials, dehydrated through a methanol (MeOH)/TN series (20%, 50%, 75%, 100% MeOH; 15 minutes each); and cleared in BABB (1 part benzyl alcohol, 2 parts benzyl benzoate) overnight at 4°C (not rocking).

### Frozen sections

Frozen sections of the injured spinal cord were difficult to obtain presumably because it contains more water than the intact cord and because the spinal column contains tissues of heterogeneous density: the muscle and bone are rigid and dense, while the spinal cord is quite soft. Therefore, methods were adapted from Tokuyasu [56]. Perfusion-fixed, decalcified tissue was cryoprotected to 2.3 M sucrose in PBS (3 hours in 25%, 3 hours in 50%, overnight in 75%, 24 hours in 100% 2.3 M sucrose; rocking and at RT) and frozen quickly in OCT (Ted Pella, Redding, CA, USA, #27050) with liquid nitrogen to minimize freezing artifacts. Sections 16 to 20 μm thick were obtained on a cryostat with the chamber temperature set to -35°C and the object temperature set to -34°C. For intact spinal cord, variables could be somewhat relaxed. Tissue could be cryoprotected to 1.6 M sucrose and perfusion was not critical. Sections were stored at -20°C and, prior to fluorescent labeling, thawed, baked onto the slides for 1 hour at 50°C, and rinsed in PBS for 5 minutes.

### Paraffin sections

All incubations were performed with rocking at RT. Decalcified tissue was dehydrated with ethanol (EtOH; 50% for 30 minutes, 70% for 30 minutes, 95% for 30 minutes, 100% for 30 minutes, 100% for 1 hour), infiltrated (75% Hemo-De in EtOH for 20 minutes, 100% Hemo-De for 1 hour, paraffin for 1 hour at 60°C and 15 Hg vacuum), and embedded in paraffin. Sections 10 to 20 μm thick were mounted onto slides, left on a 37°C slide warmer overnight, and stored at 4°C. Prior to fluorescent labeling or staining, sections were dewaxed (Hemo-De for 10 minutes, 2 times; 75% Hemo-De in EtOH for 5 minutes; 100% EtOH briefly and then for 3 minutes) and rehydrated (95% EtOH for 3 minutes, 70% EtOH for 3 minutes, PBS or water for 5 minutes; PBS was used for fluorescent labeling and water was used for staining).

### H&E staining

Paraffin sections rehydrated in water were stained: hematoxylin (Harris modified, with acetic acid; Fisher, Waltham, MA, USA, SH26-500D) 4 minutes, running tap water 5 minutes, 70% EtOH briefly, acidified 70% EtOH (10 drops of concentrated HCl to 200 ml 70% EtOH) 1.5 minutes, bluing solution (10 drops of NH_4_OH in 200 ml EtOH) 1 minute, Scott's solution (1 g sodium bicarbonate, 10 g magnesium sulfate in 500 ml water) 3 minutes, running tap water 5 minutes, 70% EtOH briefly, 0.1% Eosin in 95% EtOH (made from 0.5% Eosin Y Solution, Sigma HT110-1-32) 1 minute, 95% EtOH briefly, 100% EtOH 5 minutes, 75% Hemo-De in EtOH 5 minutes, 100% Hemo-De 5 minutes. Coverslips were mounted with Cytoseal 60 (Fisher, 23-244-256).

### Fluorescent labeling in paraffin or frozen sections

Sections were blocked in PBS-TxB (PBS with 0.2% Triton X-100, 1% BSA and 0.1% fish skin gelatin) for 1 hour; incubated with primary antibody diluted in PBS-Tx (PBS with 0.2% Triton X-100) overnight at RT; rinsed with PBS for 10 minutes, three times; incubated with secondary antibody or streptavidin diluted in PBS-Tx for 4 hours; rinsed with PBS for 10 minutes, two times; rinsed with TNT or TN for 10 minutes; incubated with SYTOX green (Invitrogen, S7020) diluted 1/1,000 in DMSO, then 1/50 in TNT or Hoechst 33342 (Invitrogen, A10027) diluted 1/2,000 in TNT for 30 minutes to stain nuclei; rinsed in TN for 10 minutes, 50% TN for 10 minutes, and water for 10 minutes. Coverslips were mounted with Fluoromount G (Fisher, OB100-01) and sealed with clear nail polish. For some experiments on paraffin sections, incubation with primary antibody was extended to 2 or 3 days at RT. This improved signal without adding noise. Sections treated with chondroitinase ABC (chABC) were treated before the blocking step. They were equilibrated in tris acetate buffer (50 mM Tris-HCl, pH 8.5, 60 mM sodium acetate, 0.02% BSA, 0.5% Triton X-100; made fresh, pH 8.0) for 5 minutes, treated with chABC solution (7.5 U/ml chABC stock diluted 1:50 in tris acetate buffer; stock was chABC, Sigma C2905, EC 4.2.2.4, reconstituted in 0.01% BSA) for 1 hour at 37°C, and rinsed with PBS for 5 minutes, three times.

### Antibodies and streptavidins

Primary antibodies and dilutions used are listed in Table 2. Additional primary antibodies that were tried are listed in Additional file 13. Secondary antibodies (Invitrogen, whole antibodies, 2 mg/ml, conjugated to Alexa 488, 568, 594, or 633) were diluted 1/100. Streptavidin-Cy5 (Invitrogen, SA1011) and Streptavidin-Alexa 633 (Invitrogen, S21375) were diluted to 4 μg/ml. The FN and Col XII antibodies and one TN-C antibody are newt-specific and have been characterized [57-59]. The non-newt-specific antibodies appear to be specific in that they produced appropriate expression patterns and are specific in western blots (not shown, not done for vWF and CSPG antibodies). The specificity of the CSPG antibody was verified by treating adjacent sections with chABC and incubating them with the CSPG antibody. The specificity of the secondary antibodies was verified by treating adjacent sections with secondary antibodies in the absence of primary antibodies. Very little non-specific signal was seen in either case (Figure 4W,X), and what little was seen tended to appear in the white matter. The chick-specific TN-C antibody produced an expression pattern similar to the newt-specific antibody, but its signal was much stronger and so it was used instead. The antibody against Col XII was used to approximate the expression of Col I since it is found in association with Col I and may enhance the stability of the ECM by bridging collagen fibrils [60,61]. Since paraffin processing may affect CS-56 labeling, CSPGs were analyzed in frozen sections. TN-C, FN, LM, and Col were analyzed in paraffin and frozen sections. Expression patterns were also verified in whole-mount preparations (not shown).

**Table 2:** Table of antibodies

Antigen	Antibody type	Company, catalogue number	Dilution, format
CSPGs (chick)	Mouse IgM mAb	Sigma, C8035 (CS-56)	1/100, ascites
Tenascin-C (chick)	Rabbit pAb	Chemicon, AB19013	1/100, concentrate
Tenascin-C (newt)	Mouse IgM mAb	DSHB, MT1	1/25, concentrate
Fibronectin (newt)	Mouse IgG mAb	DSHB, MT4	1/50, concentrate
Collagen XII (newt)	Mouse IgG1 mAb	DSHB, MT2	1/50, concentrate
Laminin (mouse)	Rabbit pAb	Sigma, L9393	1/25, concentrate
Fibrin (human)	Mouse IgG1 mAb	ADI, 350	1/50, concentrate
Neurofilament associated protein (chick)	Mouse IgG1 mAb	DSHB, 3A10	1/50, supernatant
GFAP (cow)	Rabbit pAb	Dako, Z0334	1/200, concentrate
von Willebrand factor (human)	Rabbit pAb	Dako, A0082	1/400, concentrate

ADI, American Diagnostica Inc.; DHSB, Developmental Studies Hybridoma Bank; mAb, monoclonal antibody; pAb, polyclonal antibody.

### Confocal imaging and image processing

Sections labeled with fluorescent dyes were imaged on an Olympus FV300 or FV1000 laser scanning confocal microscope using a 10× or 20× air objective. Images were processed with Image J, version 1.40 g [62] and Adobe Photoshop CS2. Levels were adjusted in Photoshop to maximize the signal to noise ratio but the same adjustments were made to experimental and control images.

### Preparation of tissue for electron microscopy

Methods were adapted from Anderson *et al*. [63]. Animals were anesthetized as above, then perfused with about 1.5 ml 85% PBS followed by 3 ml EM fixative (2.5% glutaraldehyde, 1% formaldehyde, 3% sucrose, 1 mM MgSO_4 _in 0.1 M cacodylate buffer (CB), pH 7.4). The spinal column was harvested in about 4 mm long segments; post-fixed in EM fixative overnight at RT; decalcified in cold EDTA solution (0.1 M EDTA, pH 7.2 to 7.4 with 4% glutaraldehyde) for 4 days at 4°C; rinsed in 0.1 M CB for 20 minutes, three times; rinsed in PBS briefly; and embedded in 4% agarose. Longitudinal sections containing the whole spinal cord (about 350 μm thick) were cut on a vibratome and collected in ice cold PBS. Sections were returned to 0.1 M CB; osmicated (1% OsO_4 _in 0.1 M CB) for 1 hour; rinsed in 0.1 M CB for 10 minutes, three times; partially dehydrated (50%, 75% MeOH; 10 minutes each); treated with 1% uranyl acetate in 75% MeOH for 1 hour; fully dehydrated (75%, 85%, 95%, 100%, 100% MeOH; 10 minutes each); infiltrated with resin (100% acetone 10 minutes, two times; 75% resin in acetone overnight; 100% resin 1 hour); embedded in epoxy resin (2 parts dodecenyl succinic anhydride (DDSA, Ted Pella, 18022), 1 part eponate 12 resin (Ted Pella, 18005), 0.06 parts DMP30 accelerator (Ted Pella, 18042)) and cured at 60°C for 2 days. Sections were re-embedded for longitudinal sectioning, and 80-nm sections were collected onto carbon-coated Formvar films in gold single-hole grids, stained with 5% uranyl acetate for 1 hour and 1% lead citrate for 25 minutes, and imaged at 80 KeV in a JEOL JEM 1400 electron microscope at 5,000× magnification. Images were captured digitally on a GATAN Ultrascan 4000 16 megapixel 16-bit camera and mosaicked with *ir-tools *[63]. Mosaics were viewed and selections were captured with Viking software.

## Abbreviations

BDA: biotinylated dextran amine; BSA: bovine serum albumin; CB: cacodylate buffer; chABC: chondroitinase ABC; Col: collagen; CSPG: chondroitin sulfate proteoglycan; DMSO: dimethylsulfoxide; ECM: extracellular matrix; EG: ependymoglia; EM: electron microscopy; EtOH: ethanol; FN: fibronectin; GFAP: glial fibrillary acidic protein; H&E: hematoxylin and eosin; LM: laminin; MeOH: methanol; OEC: olfactory ensheathing cell; PBS: phosphate buffered saline; PFA: paraformaldehyde; PLP: periodate-lysine-PFA; RDA: rhodamine dextran amine; RT: room temperature; SCI: spinal cord injury; TN-C: tenascin C; TV: terminal vesicle; Tx: Triton X-100; vWF: von Willebrand factor.

## Competing interests

The authors declare that they have no competing interests.

## Authors' contributions

KAZ conceived of the study with the assistance of SJO, designed and conducted the experiments, analyzed the data, prepared the figures and wrote the manuscript. DTK and SJO helped design the study and experiments, helped interpret the data, and provided comments on the manuscript. SJO is the laboratory principal investigator and oversaw the project. All authors read and approved the final manuscript.

## Supplementary Material

Additional file 1 **Movie 1: recovery of swimming function after spinal cord transection injury**. When an uninjured newt swims, it propels itself forward by pressing its legs close to its side and undulating its body in an S-shaped motion. When it is finished swimming, it brings its legs forward again, perpendicular to its body axis. One day after a complete transection injury, the hindlimbs are completely paralyzed. Four weeks after injury, this same newt is still paralyzed. When it attempts to swim, it does not use its hindlimbs at all. By 7 weeks, this newt has recovered swimming function and swims similarly to an uninjured newt. Click here for file

Additional file 2 **Figure S1: the largest spinal nerves are associated with the S1 and T-1 vertebrae**. **(A) **Close-up view of S1, dorsal aspect. The rib associated with this vertebra articulates with the ilium of the pelvis to form the SI joint. **(B) **Ventral side of spinal column showing vertebrae T-4 to S2/C1. The largest spinal nerves (arrowheads) are associated with T-1 and S1. T-2 is intermediate in size. T-4, T-3 and S2/C1 are small. Dotted circles, approximate location of the spinal ganglia; dotted lines, approximate course spinal nerves take to spinal ganglia. Note that the actual location of the SCI in this animal is one segment rostral (between T-5 and T-4) to the targeted site (between T-4 and T-3). **(C) **Close-up of T-1 and S1 shown in (B). More flesh has been removed to demonstrate that the vertebra associated with the caudal-most large spinal nerve is indeed S1. The rib associated with it articulated with the pelvis. R, rostral; C, caudal. Click here for file

Additional file 3 **Figure S2: retraction and growth initiation stage images seen in z-projections**. The difference between the retraction and growth initiation stages is apparent throughout the whole spinal cord and is not just a function of which z-plane was chosen for presentation. **(A-D) **Images of retraction (A, B) and growth initiation (C, D) shown in Figure 2. All are single confocal planes, except (B), which is a z-projection of four planes. **(A'-D') **Z-projections of all planes through the spinal cord, showing just the axon tracer channel, for the same animals in (A-D). In the retraction stage, fewer axons extend to the end of the cut cord (arrowheads). R, rostral; C, caudal. Scale bars: 200 μm ((A-D) are the same scale; (A'-D') are the same scale). Click here for file

Additional file 4 **Figure S3: size of gap in spinal cord at each stage**. Gap size increases before it decreases and is largest during the wrapping stage. Dark lines, median; box, interquartile range (IQR, 25% to 75%); whiskers, most extreme data point that is no more than 1.5 IQR from the box; small circle, outlier. The size of the gap during the wrapping stage is statistically different (asterisk) from that during the retraction stage, with *P *< 0.001 (using Bonferroni correction for multiple *t*-tests). Click here for file

Additional file 5 **Movie 2: movie through confocal z-stack of animal shown in **Figure 5D**, a wisping/spiking stage regenerate**. The movie begins on the ventral side of the cord and moves in 2-μm increments through to the dorsal side. Rostral is up. Axons are labeled with 3A10 in magenta, and nuclei are in green. Note the meninges appear to have regenerated across the gap first and that some axons appear to have followed these meninges. Click here for file

Additional file 6 **Movie 3: movie through confocal z-stack of animal shown in **Figure 2J**, a spiking stage regenerate**. The spike does not contain an ependymal tube in this animal. The movie begins on the ventral side of the cord and moves in 2-μm increments through to the dorsal side. Rostral is up. Descending axons are labeled with the axon tracer in magenta and nuclei are in green. Click here for file

Additional file 7 **Movie 4: movie through confocal z-stack of animal shown in **Figure 2I**, a spiking stage regenerate**. The spike does contain an ependymal tube in this animal. The movie begins on the ventral side of the cord and moves in 2-μm increments through to the dorsal side. Rostral is up. Descending axons are labeled with the axon tracer in magenta and nuclei are in green. Click here for file

Additional file 8 **Figure S4: sloppiness and misalignments in axon regeneration**. Axon regeneration has some tolerance for sloppiness, but gross misalignments may result in a delay or failure to recover. All images are montages of single confocal planes of longitudinal thick sections, except (F) which is a cross-section. Axons in all images were labeled with the axon tracer (magenta), and nuclei are green. **(A-A") **A 9-week regenerate in the growth beyond stage that had recovered swimming function. The ependymal tubes have connected (A), some axons appear to decussate (arrowhead in (A')) and wrapping axons are still evident (open arrowhead in (A")). **(B) **A 4-week regenerate in the growth beyond stage that had not recovered function. Similar to (A), axons appear to wrap (open arrowhead) and decussate (arrowhead). **(C) **A 9-week regenerate in the growth beyond stage that had not recovered function. A long spike appears to have elongated from the caudal side (dotted lines), and this may indicate that the gap in the cord was large. **(D) **A 6-week regenerate in the spiking stage that had not recovered function. The whole spike appears to be veering off in the wrong direction and does not line up properly with the cord on the other side (dotted lines). Arrow, dorsal root to the spinal ganglia. **(E) **A 9-week regenerate in the growth beyond stage that had not recovered function. Two ependymal tubes (fat arrows) appear to have formed on the rostral side. **(F) **Cross-section through a 9-week regenerate in the growth beyond stage that had not recovered function. Axon regeneration appears to be sloppy in that axons are wisping dorsally towards the tissue wound that was created to gain access to the spinal cord. R, rostral; C, caudal; D, dorsal; V, ventral. Scale bars: 500 μm ((A-E) are the same scale); 200 μm (F). Click here for file

Additional file 9 **Movie 5: movie through confocal z-stack of animal shown in Additional file **8** A-A", a recovered animal**. This is a 9-week regenerate in the growth beyond stage that had recovered function. The movie begins on the ventral side of the cord and moves in 2-μm increments through to the dorsal side. Rostral is up. Descending axons are labeled with the axon tracer in magenta and nuclei are in green. Click here for file

Additional file 10 **Movie 6: movie through confocal z-stack of animal shown in Additional file **8E, **a non-recovered animal**. This is a 9-week regenerate in the growth beyond stage that had not recovered function. It begins on the ventral side of the cord and moves in 2-μm increments through to the dorsal side. Rostral is up. Descending axons are labeled with the axon tracer in magenta and nuclei are in green. Note there are two ependymal tubes on the rostral side. Click here for file

Additional file 11 Table S1: Stages of 2.5- to 3-week regenerates analyzed after initial study. Click here for file

Additional file 12 **Figure S5: a glia limitans-like structure may be present during the wrapping stage**. Longitudinal sections through wrapping stage regenerates. Axons were labeled with the axon tracer in (C, D) and are shown in magenta. Each ECM protein is shown in green, and nuclei are blue. **(A-D) **Col XII (A), FN (B), and TN-C (C) expression wraps around the end of the cord, while LM (D) expression does not. R, rostral; C, caudal. Scale bar: 200 μm (A-D). Click here for file

Additional file 13 Table S2: Table of other antibodies tested. Click here for file

Additional file 14 **Figure S6: meninges of the intact spinal cord**. Longitudinal section through the intact spinal cord imaged with EM. **(A) **Region containing the meninges. p, pia mater; a, arachnoid mater; d, dura mater; sas, subarachnoid space; ecm, ECM; ef, glial end feet; o, oligodendrocyte. **(B) **Enlargement of box B in (A) showing skinny, dark processes of dura mater cells (arrows) associated with collagen fibrils (c) of the dura mater. **(C) **Enlargement of box C in (A) showing layers of arachnoid cell processes (a), a skinny process from a dura mater cell (arrow), and collagen (c). **(D) **Enlargement of box D in (A) showing a pia mater cell. **(E) **Enlargement of box E in (A) showing neural collagen (nc). **(F) **Enlargement of box F in (A) showing the basement membrane (arrowhead) at the glia limitans of the spinal cord, collagen (c) and neural collagen (nc). R, rostral; C, caudal. Scale bars: 15 μm (A); 3 μm (D); 1.5 μm (B, C, E, F). Click here for file

Additional file 15 **Figure S7: other cell types in the intact spinal cord**. Longitudinal section through the intact spinal cord imaged with EM. **(A) **Region containing the central canal (cc), EG layer (EG) and a portion of the grey mater (gm). **(B) **Enlargement of box B in (A) showing astrocytes. **(C) **Enlargement of box C in (A) showing the cytoplasm of light EG. **(D) **Enlargement of box D in (A) showing the cytoplasm of dark EG. **(E) **A microglial cell. **(F) **An oligodendrocyte. R, rostral; C, caudal. Scale bars: 15 μm (A, B, F); 5 μm (E); 3 μm (D); 1.5 μm (C). Click here for file

Additional file 16 **Figure S8: glia of the regenerating spinal cord**. (**A-D**) Longitudinal section through a wisping stage regenerate imaged with EM. **(A) **Region containing axons wisping ahead of the TV. **(B) **Enlargement of box B in (A) showing a light glial process (EG_L_) that is associated with regenerating axons (ax). **(C) **Enlargement of box C in (A) showing light (EG_L_) and dark (EG_D_) EG lining the TV. **(D) **Enlargement of box D in (A) showing a dark EG process (EG_D_) associated with axons (ax). R, rostral; C, caudal. Scale bars: 50 μm (A); 15 μm (C); 5 μm (B); 3 μm (D). Click here for file

Additional file 17 **Figure S9: the inflammatory response in early and late stage regenerates**. **(A,B**) Adjacent longitudinal sections through a 1-week regenerate labeled with an anti-fibrin antibody (A) and stained with H&E (B). A fibrin clot is formed in the injury site (A), and inflammatory cells can already be identified in this clot (B). **(B',B") **Enlargement of primed and double primed boxes in (B). Lymphocytes (arrowheads in (B')) and monocytes (arrow in (B")) can be identified. Asterisk, fibrin clot. **(C) **Longitudinal section through a contact stage regenerate. A relatively strong inflammatory response has not prevented this animal from progressing to this late stage. **(C') **Enlargement of box in (C). Lymphocytes (arrowhead) and monocytes (arrows) can be identified. R, rostral; C, caudal. Scale bars: 200 μm (A; (B,C) are the same scale); 50 μm ((B',B") are the same scale; C'). Click here for file

## References

[B1] Davis BM Ayers JL Koran L Carlson J Anderson MC Simpson SB Jr Time course of salamander spinal cord regeneration and recovery of swimming: HRP retrograde pathway tracing and kinematic analysis Exp Neurol 1990 108 198 213 10.1016/0014-4886(90)90124-B 2351209

[B2] Silver J Miller JH Regeneration beyond the glial scar Nat Rev Neurosci 2004 5 146 156 10.1038/nrn1326 14735117

[B3] Singer M Nordlander RH Egar M Axonal guidance during embryogenesis and regeneration in the spinal cord of the newt: the blueprint hypothesis of neuronal pathway patterning J Comp Neurol 1979 185 1 21 10.1002/cne.901850102 429610

[B4] Piatt J Regeneration of the spinal cord in the salamander J Exp Zool 1955 129 177 207 10.1002/jez.1401290109

[B5] Butler EG Ward MB Reconstitution of the spinal cord after ablation in adult *Triturus* Dev Biol 1967 15 464 486 10.1016/0012-1606(67)90038-3 6032488

[B6] Stensaas LJ Kao CC, Bunge RP, Reier PJ Regeneration in the spinal cord of the newt *Notopthalamus (Triturus) pyrrhogaster* Spinal Cord Reconstruction 1983 New York: Raven Press 121 149

[B7] O'Hara CM Egar MW Chernoff EA Reorganization of the ependyma during axolotl spinal cord regeneration: changes in intermediate filament and fibronectin expression Dev Dyn 1992 193 103 115 1374657 10.1002/aja.1001930202

[B8] Chernoff EA Stocum DL Nye HL Cameron JA Urodele spinal cord regeneration and related processes Dev Dyn 2003 226 295 307 10.1002/dvdy.10240 12557207

[B9] Shearer MC Fawcett JW The astrocyte/meningeal cell interface - a barrier to successful nerve regeneration? Cell Tissue Res 2001 305 267 273 10.1007/s004410100384 11545264

[B10] Bundesen LQ Scheel TA Bregman BS Kromer LF Ephrin-B2 and EphB2 regulation of astrocyte-meningeal fibroblast interactions in response to spinal cord lesions in adult rats J Neurosci 2003 23 7789 7800 12944508 10.1523/JNEUROSCI.23-21-07789.2003 PMC6740614

[B11] Francis ETB The Anatomy of the Salamander 2002 Salt Lake City: Society for the Study of Amphibians and Reptiles

[B12] Davis BM Duffy MT Simpson SB Jr Bulbospinal and intraspinal connections in normal and regenerated salamander spinal cord Exp Neurol 1989 103 41 51 10.1016/0014-4886(89)90183-0 2912749

[B13] Ramony Cajal S Degeneration and Regeneration of the Nervous System 1928 London: Oxford University Press

[B14] Tom VJ Steinmetz MP Miller JH Doller CM Silver J Studies on the development and behavior of the dystrophic growth cone, the hallmark of regeneration failure, in an *in vitro *model of the glial scar and after spinal cord injury J Neurosci 2004 24 6531 6539 10.1523/JNEUROSCI.0994-04.2004 15269264 PMC6729861

[B15] Becker T Wullimann MF Becker CG Bernhardt RR Schachner M Axonal regrowth after spinal cord transection in adult zebrafish J Comp Neurol 1997 377 577 595 10.1002/(SICI)1096-9861(19970127)377:4<577::AID-CNE8>3.0.CO;2-# 9007194

[B16] Becker T Becker CG Regenerating descending axons preferentially reroute to the gray matter in the presence of a general macrophage/microglial reaction caudal to a spinal transection in adult zebrafish J Comp Neurol 2001 433 131 147 10.1002/cne.1131 11283955

[B17] Xie F Zheng B White matter inhibitors in CNS axon regeneration failure Exp Neurol 2008 209 302 312 10.1016/j.expneurol.2007.07.005 17706966 PMC2259386

[B18] Tang X Davies JE Davies SJ Changes in distribution, cell associations, and protein expression levels of NG2, neurocan, phosphacan, brevican, versican V2, and tenascin-C during acute to chronic maturation of spinal cord scar tissue J Neurosci Res 2003 71 427 444 10.1002/jnr.10523 12526031

[B19] Davies SJ Goucher DR Doller C Silver J Robust regeneration of adult sensory axons in degenerating white matter of the adult rat spinal cord J Neurosci 1999 19 5810 5822 10407022 10.1523/JNEUROSCI.19-14-05810.1999 PMC6783087

[B20] Herrmann JE Shah RR Chan AF Zheng B EphA4 deficient mice maintain astroglial-fibrotic scar formation after spinal cord injury Exp Neurol 2010 223 582 598 10.1016/j.expneurol.2010.02.005 20170651 PMC2864333

[B21] Busch SA Horn KP Cuascut FX Hawthorne AL Bai L Miller RH Silver J Adult NG2+ cells are permissive to neurite outgrowth and stabilize sensory axons during macrophage-induced axonal dieback after spinal cord injury J Neurosci 2010 30 255 265 10.1523/JNEUROSCI.3705-09.2010 20053907 PMC2823089

[B22] Hermanns S Reiprich P Muller HW A reliable method to reduce collagen scar formation in the lesioned rat spinal cord J Neurosci Methods 2001 110 141 146 10.1016/S0165-0270(01)00427-7 11564534

[B23] Condic ML Lemons ML Extracellular matrix in spinal cord regeneration: getting beyond attraction and inhibition Neuroreport 2002 13 A37 48 10.1097/00001756-200203040-00002 11930141

[B24] McKeon RJ Hoke A Silver J Injury-induced proteoglycans inhibit the potential for laminin-mediated axon growth on astrocytic scars Exp Neurol 1995 136 32 43 10.1006/exnr.1995.1081 7589332

[B25] Caubit X Riou JF Coulon J Arsanto JP Benraiss A Boucaut JC Thouveny Y Tenascin expression in developing, adult and regenerating caudal spinal cord in the urodele amphibians Int J Dev Biol 1994 38 661 672 7540033

[B26] Larrivee B Freitas C Suchting S Brunet I Eichmann A Guidance of vascular development: lessons from the nervous system Circ Res 2009 104 428 441 10.1161/CIRCRESAHA.108.188144 19246687

[B27] Mey J D JM Brook G Liu RH Zhang YP Koopmans G McCaffery P Retinoic acid synthesis by a population of NG2-positive cells in the injured spinal cord Eur J Neurosci 2005 21 1555 1568 10.1111/j.1460-9568.2005.03928.x 15845083

[B28] Li Y Field PM Raisman G Regeneration of adult rat corticospinal axons induced by transplanted olfactory ensheathing cells J Neurosci 1998 18 10514 10524 9852589 10.1523/JNEUROSCI.18-24-10514.1998 PMC6793366

[B29] Lakatos A Smith PM Barnett SC Franklin RJ Meningeal cells enhance limited CNS remyelination by transplanted olfactory ensheathing cells Brain 2003 126 598 609 10.1093/brain/awg055 12566281

[B30] Harty M Neff AW King MW Mescher AL Regeneration or scarring: an immunologic perspective Dev Dyn 2003 226 268 279 10.1002/dvdy.10239 12557205

[B31] Fitch MT Doller C Combs CK Landreth GE Silver J Cellular and molecular mechanisms of glial scarring and progressive cavitation: *in vivo *and *in vitro *analysis of inflammation-induced secondary injury after CNS trauma J Neurosci 1999 19 8182 8198 10493720 10.1523/JNEUROSCI.19-19-08182.1999 PMC6783021

[B32] Horn KP Busch SA Hawthorne AL van Rooijen N Silver J Another barrier to regeneration in the CNS: activated macrophages induce extensive retraction of dystrophic axons through direct physical interactions J Neurosci 2008 28 9330 9341 10.1523/JNEUROSCI.2488-08.2008 18799667 PMC2567141

[B33] Donnelly DJ Popovich PG Inflammation and its role in neuroprotection, axonal regeneration and functional recovery after spinal cord injury Exp Neurol 2008 209 378 388 10.1016/j.expneurol.2007.06.009 17662717 PMC2692462

[B34] Simpson SB Jr Kao CC, Bunge RP, Reier PJ Fasciculation and guidance of regenerating central axons by the ependyma Spinal Cord Reconstruction 1983 New York: Raven Press 151 162

[B35] Schonbach C The neuroglia in the spinal cord of the newt, *Triturus viridescens* J Comp Neurol 1969 135 93 120 10.1002/cne.901350107 5780525

[B36] Ghashghaei HT Lai C Anton ES Neuronal migration in the adult brain: are we there yet? Nat Rev Neurosci 2007 8 141 151 10.1038/nrn2074 17237805

[B37] Ferguson MW O'Kane S Scar-free healing: from embryonic mechanisms to adult therapeutic intervention Philos Trans R Soc Lond B Biol Sci 2004 359 839 850 10.1098/rstb.2004.1475 15293811 PMC1693363

[B38] Borrell V Marin O Meninges control tangential migration of hem-derived Cajal-Retzius cells via CXCL12/CXCR4 signaling Nat Neurosci 2006 9 1284 1293 10.1038/nn1764 16964252

[B39] Siegenthaler JA Ashique AM Zarbalis K Patterson KP Hecht JH Kane MA Folias AE Choe Y May SR Kume T Napoli JL Peterson AS Pleasure SJ Retinoic acid from the meninges regulates cortical neuron generation Cell 2009 139 597 609 10.1016/j.cell.2009.10.004 19879845 PMC2772834

[B40] Shearer MC Niclou SP Brown D Asher RA Holtmaat AJ Levine JM Verhaagen J Fawcett JW The astrocyte/meningeal cell interface is a barrier to neurite outgrowth which can be overcome by manipulation of inhibitory molecules or axonal signalling pathways Mol Cell Neurosci 2003 24 913 925 10.1016/j.mcn.2003.09.004 14697658

[B41] Clagett-Dame M McNeill EM Muley PD Role of all-trans retinoic acid in neurite outgrowth and axonal elongation J Neurobiol 2006 66 739 756 10.1002/neu.20241 16688769

[B42] Wang G Scott SA Retinoid signaling is involved in governing the waiting period for axons in chick hindlimb Dev Biol 2008 321 216 226 10.1016/j.ydbio.2008.06.021 18602384 PMC2596718

[B43] Becker CG Becker T Meyer RL Schachner M Tenascin-R inhibits the growth of optic fibers *in vitro *but is rapidly eliminated during nerve regeneration in the salamander Pleurodeles waltl J Neurosci 1999 19 813 827 9880601 10.1523/JNEUROSCI.19-02-00813.1999 PMC6782211

[B44] Condic ML Snow DM Letourneau PC Embryonic neurons adapt to the inhibitory proteoglycan aggercan by increasing integrin expression J Neurosci 1999 19 10036 10043 10559411 10.1523/JNEUROSCI.19-22-10036.1999 PMC6782993

[B45] Lemons ML Barua S Abanto ML Halfter W Condic ML Adaptation of sensory neurons to hyalectin and decorin proteoglycans J Neurosci 2005 25 4964 4973 10.1523/JNEUROSCI.0773-05.2005 15901777 PMC6724852

[B46] Kumar A Godwin JW Gates PB Garza-Garcia AA Brockes JP Molecular basis for the nerve dependence of limb regeneration in an adult vertebrate Science 2007 318 772 777 10.1126/science.1147710 17975060 PMC2696928

[B47] Tennant M Beazley LD A breakdown of the blood-brain barrier is associated with optic nerve regeneration in the frog Vis Neurosci 1992 9 149 155 10.1017/S0952523800009615 1504024

[B48] Reier PJ Stensaas LJ Guth L Kao CC, Bunge RP, Reier PJ The astrocytic scar as an impediment to regeneration in the central nervous system Spinal Cord Reconstruction 1983 New York: Raven Press 163 196

[B49] Ferretti P Zhang F O'Neill P Changes in spinal cord regenerative ability through phylogenesis and development: lessons to be learnt Dev Dyn 2003 226 245 256 10.1002/dvdy.10226 12557203

[B50] Odelberg SJ Cellular plasticity in vertebrate regeneration Anat Rec B New Anat 2005 287 25 35 16308861 10.1002/ar.b.20080

[B51] Liu K Lu Y Lee JK Samara R Willenberg R Sears-Kraxberger I Tedeschi A Park KK Jin D Cai B Xu B Connolly L Steward O Zheng B He Z PTEN deletion enhances the regenerative ability of adult corticospinal neurons Nat Neurosci 2010 13 1075 1081 10.1038/nn.2603 20694004 PMC2928871

[B52] McLean IW Nakane PK Periodate-lysine-paraformaldehyde fixative. A new fixation for immunoelectron microscopy J Histochem Cytochem 1974 22 1077 1083 4374474 10.1177/22.12.1077

[B53] Kardon G Muscle and tendon morphogenesis in the avian hind limb Development 1998 125 4019 4032 9735363 10.1242/dev.125.20.4019

[B54] Klymkowsky MW Hanken J Whole-mount staining of Xenopus and other vertebrates Methods in Cell Biology 1991 36 Academic Press, Inc 419 441 full_text 1725802 10.1016/s0091-679x(08)60290-3

[B55] Zukor KA Kent DT Odelberg SJ Fluorescent whole-mount method for visualizing 3-dimensional relationships in intact and regenerating adult newt spinal cords Dev Dyn in press 20931649 10.1002/dvdy.22441 PMC3013515

[B56] Tokuyasu KT Use of poly(vinylpyrrolidone) and poly(vinyl alcohol) for cryoultramicrotomy Histochem J 1989 21 163 171 10.1007/BF01007491 2722561

[B57] Wei Y Yang EV Klatt KP Tassava RA Monoclonal antibody MT2 identifies the urodele alpha 1 chain of type XII collagen, a developmentally regulated extracellular matrix protein in regenerating newt limbs Dev Biol 1995 168 503 513 10.1006/dbio.1995.1098 7729585

[B58] Onda H Goldhamer DJ Tassava RA An extracellular matrix molecule of newt and axolotl regenerating limb blastemas and embryonic limb buds: immunological relationship of MT1 antigen with tenascin Development 1990 108 657 668 1696876 10.1242/dev.108.4.657

[B59] Nace JD Tassava RA Examination of fibronectin distribution and its sources in the regenerating newt limb by immunocytochemistry and in situ hybridization Dev Dyn 1995 202 153 164 7734733 10.1002/aja.1002020207

[B60] Bader HL Keene DR Charvet B Veit G Driever W Koch M Ruggiero F Zebrafish collagen XII is present in embryonic connective tissue sheaths (fascia) and basement membranes Matrix Biol 2009 28 32 43 10.1016/j.matbio.2008.09.580 18983916

[B61] Reichenberger E Baur S Sukotjo C Olsen BR Karimbux NY Nishimura I Collagen XII mutation disrupts matrix structure of periodontal ligament and skin J Dent Res 2000 79 1962 1968 10.1177/00220345000790120701 11201046

[B62] ImageJ http://rsb.info.nih.gov/ij/

[B63] Anderson JR Jones BW Yang JH Shaw MV Watt CB Koshevoy P Spaltenstein J Jurrus EUVK Whitaker RT Mastronarde D Tasdizen T Marc RE A computational framework for ultrastructural mapping of neural circuitry PLoS Biol 2009 7 e1000074 10.1371/journal.pbio.1000074 19855814 PMC2661966

