# Supplementary material for: Meningeal cells and glia establish a permissive environment for axon regeneration after spinal cord injury in newts
Source: Neural Dev. 2011 Jan 4;6:1. doi: 10.1186/1749-8104-6-1 (PMC3025934; doi:10.1186/1749-8104-6-1)
Supplement: Additional file 2 — Figure S1: the largest spinal nerves are associated with the S1 and T-1 vertebrae. (A) Close-up view of S1, dorsal aspect. The rib associated with this vertebra articulates with the ilium of the pelvis to form the SI joint. (B) Ventral side of spinal column showing vertebrae T-4 to S2/C1. The largest spinal nerves (arrowheads) are associated with T-1 and S1. T-2 is intermediate in size. T-4, T-3 and S2/C1 are small. Dotted circles, approximate location of the spinal ganglia; dotted lines, approximate course spinal nerves take to spinal ganglia. Note that the actual location of the SCI in this animal is one segment rostral (between T-5 and T-4) to the targeted site (between T-4 and T-3). (C) Close-up of T-1 and S1 shown in (B). More flesh has been removed to demonstrate that the vertebra associated with the caudal-most large spinal nerve is indeed S1. The rib associated with it articulated with the pelvis. R, rostral; C, caudal. [file 1749-8104-6-1-S2.PDF]

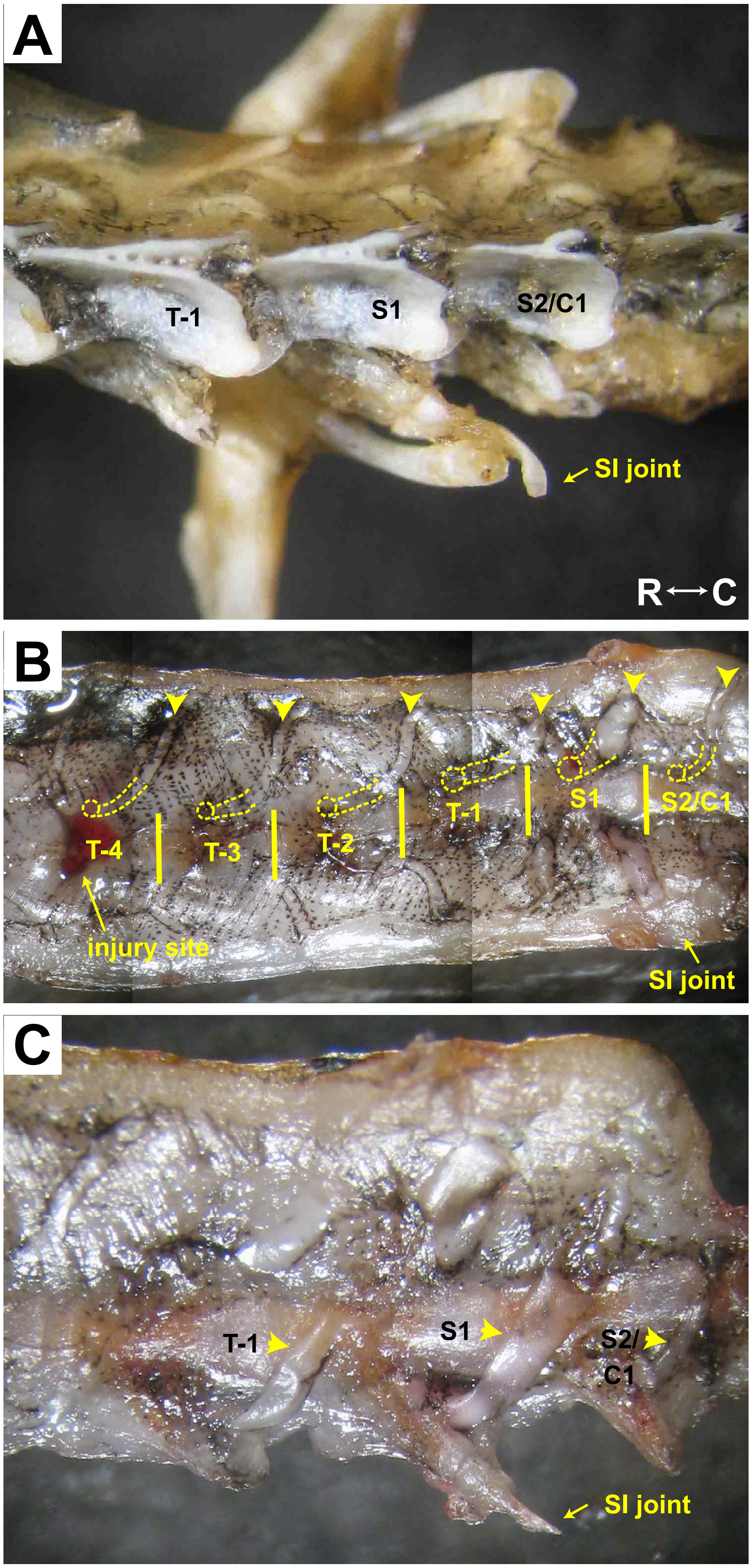

Additional file 2: Figure S1. The largest spinal nerves are associated with the S1 and T-1 vertebrae. **(A)** Close-up view of S1, dorsal aspect. The rib associated with this vertebra articulates with the ilium of the pelvis to form the SI joint. **(B)** Ventral side of spinal column showing vertebrae T-4 to S2/C1. The largest spinal nerves (arrowheads) are associated with T-1 and S1. T-2 is intermediate in size. T-4, T-3 and S2/C1 are small. Dotted circles, approximate location of the spinal ganglia; dotted lines, approximate course spinal nerves take to spinal ganglia. Note that the actual location of the SCI in this animal is one segment rostral (between T-5 and T-4) to the targeted site (between T-4 and T-3). **(C)** Close-up of T-1 and S1 shown in (B). More flesh has been removed to demonstrate that the vertebra associated with the caudal-most large spinal nerve is indeed S1. The rib associated with it articulated with the pelvis. R, rostral; C, caudal.
