# Supplementary material for: Meningeal cells and glia establish a permissive environment for axon regeneration after spinal cord injury in newts
Source: Neural Dev. 2011 Jan 4;6:1. doi: 10.1186/1749-8104-6-1 (PMC3025934; doi:10.1186/1749-8104-6-1)
Supplement: Additional file 3 — Figure S2: retraction and growth initiation stage images seen in z-projections. The difference between the retraction and growth initiation stages is apparent throughout the whole spinal cord and is not just a function of which z-plane was chosen for presentation. (A-D) Images of retraction (A, B) and growth initiation (C, D) shown in Figure 2. All are single confocal planes, except (B), which is a z-projection of four planes. (A'-D') Z-projections of all planes through the spinal cord, showing just the axon tracer channel, for the same animals in (A-D). In the retraction stage, fewer axons extend to the end of the cut cord (arrowheads). R, rostral; C, caudal. Scale bars: 200 μm ((A-D) are the same scale; (A'-D') are the same scale). [file 1749-8104-6-1-S3.PDF]

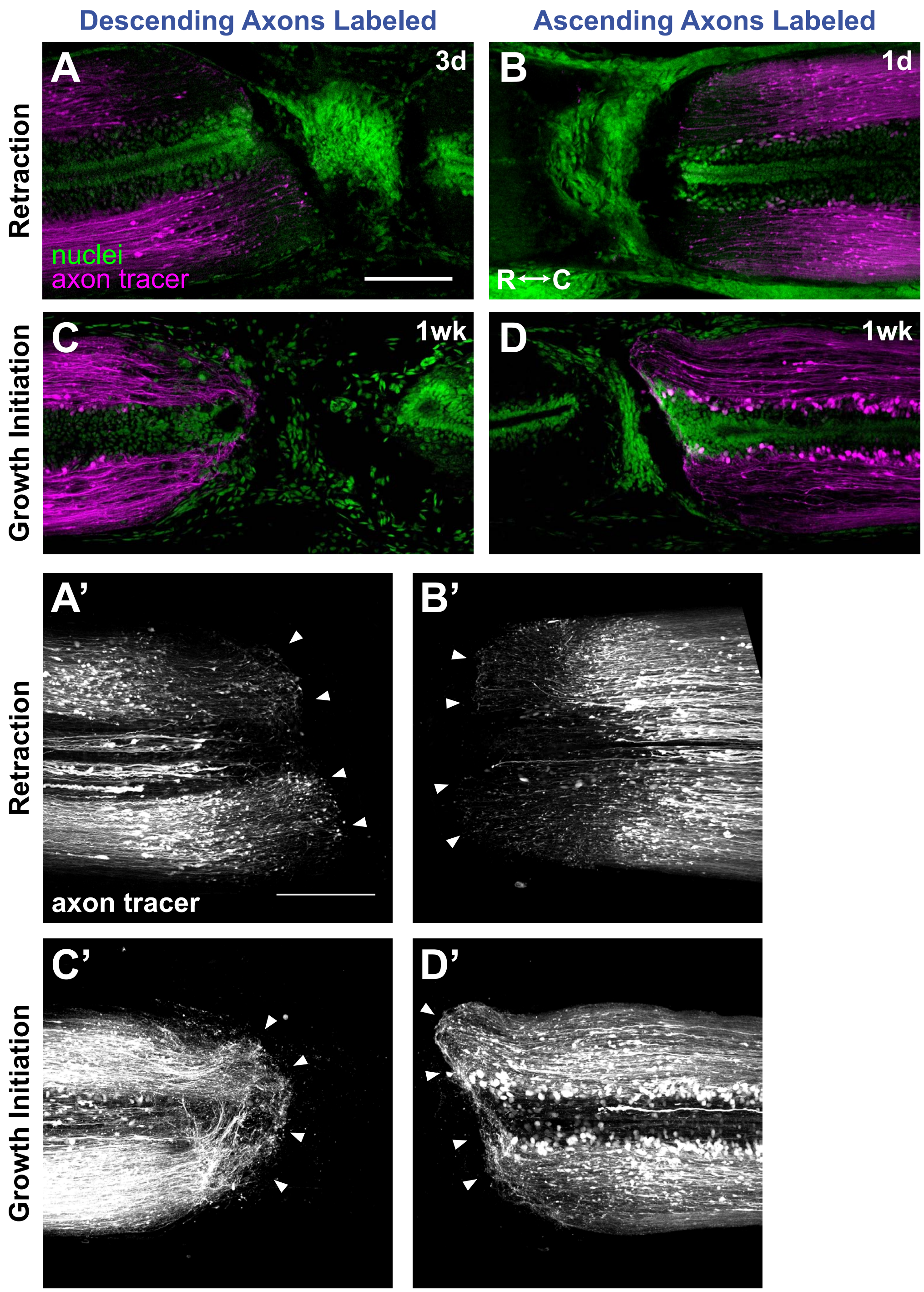

Additional file 3: Figure S2. Retraction and growth initiation stage images seen in z-projections. The difference between the retraction and growth initiation stages is apparent throughout the whole spinal cord and is not just a function of which z-plane was chosen for presentation. **(A-D)** Images of retraction (A,B) and growth initiation (C,D) shown in Figure 2. All are single confocal planes, except (B), which is a z-projection of four planes. **(A'-D')** Z-projections of all planes through the spinal cord, showing just the axon tracer channel, for the same animals in (A-D). In the retraction stage, fewer axons extend to the end of the cut cord (arrowheads). R, rostral; C, caudal. Scale bars: 200 μm ((A-D) are the same scale; (A'-D') are the same scale).
