# Supplementary material for: Meningeal cells and glia establish a permissive environment for axon regeneration after spinal cord injury in newts
Source: Neural Dev. 2011 Jan 4;6:1. doi: 10.1186/1749-8104-6-1 (PMC3025934; doi:10.1186/1749-8104-6-1)
Supplement: Additional file 4 — Figure S3: size of gap in spinal cord at each stage. Gap size increases before it decreases and is largest during the wrapping stage. Dark lines, median; box, interquartile range (IQR, 25% to 75%); whiskers, most extreme data point that is no more than 1.5 IQR from the box; small circle, outlier. The size of the gap during the wrapping stage is statistically different (asterisk) from that during the retraction stage, with P < 0.001 (using Bonferroni correction for multiple t-tests). [file 1749-8104-6-1-S4.PDF]

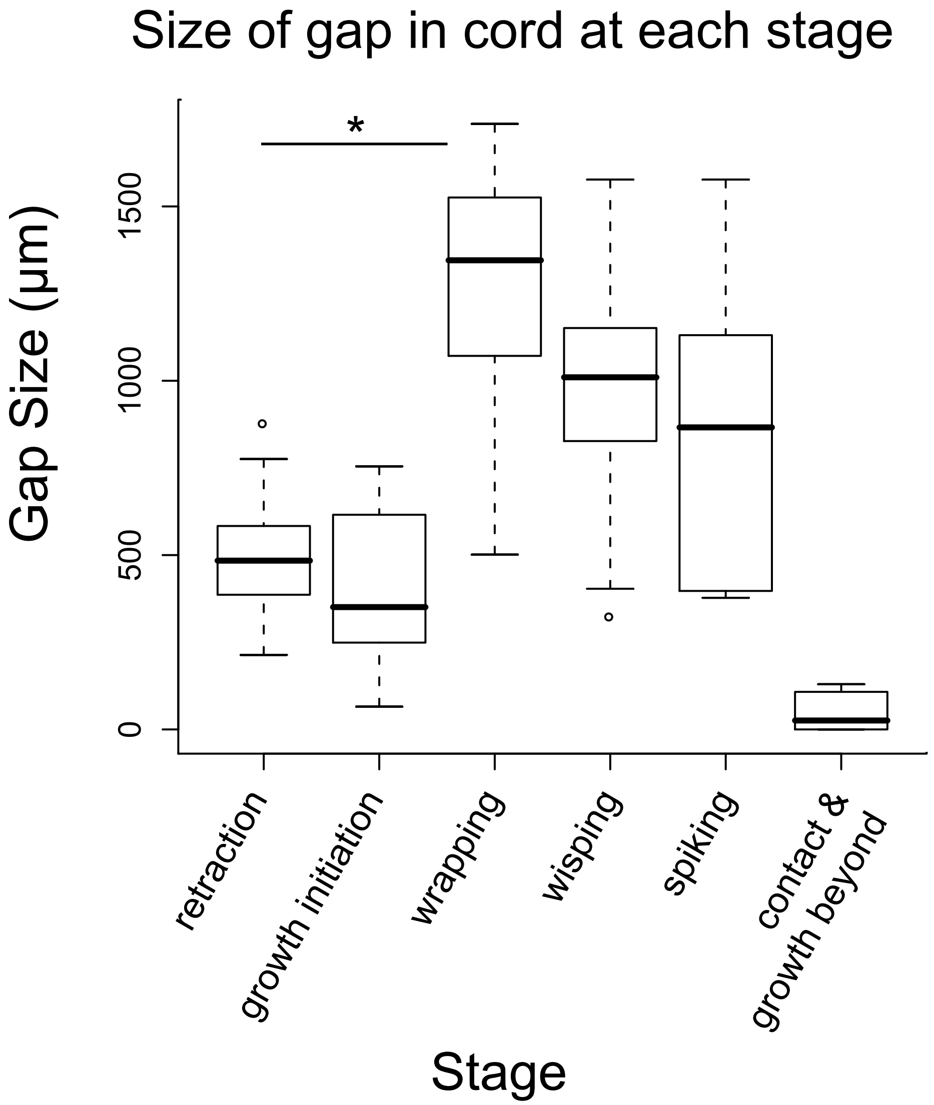

Additional file 4: Figure S3. Size of gap in spinal cord at each stage. Gap size increases before it decreases and is largest during the wrapping stage. Dark lines, median; box, interquartile range (IQR, 25% to 75%); whiskers, most extreme data point that is no more than 1.5 IQR from the box; small circle, outlier. The size of the gap during the wrapping stage is statistically different (asterisk) from that during the retraction stage, with $P < 0.001$ (using Bonferroni correction for multiple *t*-tests).
