# Supplementary material for: Meningeal cells and glia establish a permissive environment for axon regeneration after spinal cord injury in newts
Source: Neural Dev. 2011 Jan 4;6:1. doi: 10.1186/1749-8104-6-1 (PMC3025934; doi:10.1186/1749-8104-6-1)
Supplement: Additional file 8 — Figure S4: sloppiness and misalignments in axon regeneration. Axon regeneration has some tolerance for sloppiness, but gross misalignments may result in a delay or failure to recover. All images are montages of single confocal planes of longitudinal thick sections, except (F) which is a cross-section. Axons in all images were labeled with the axon tracer (magenta), and nuclei are green. (A-A") A 9-week regenerate in the growth beyond stage that had recovered swimming function. The ependymal tubes have connected (A), some axons appear to decussate (arrowhead in (A')) and wrapping axons are still evident (open arrowhead in (A")). (B) A 4-week regenerate in the growth beyond stage that had not recovered function. Similar to (A), axons appear to wrap (open arrowhead) and decussate (arrowhead). (C) A 9-week regenerate in the growth beyond stage that had not recovered function. A long spike appears to have elongated from the caudal side (dotted lines), and this may indicate that the gap in the cord was large. (D) A 6-week regenerate in the spiking stage that had not recovered function. The whole spike appears to be veering off in the wrong direction and does not line up properly with the cord on the other side (dotted lines). Arrow, dorsal root to the spinal ganglia. (E) A 9-week regenerate in the growth beyond stage that had not recovered function. Two ependymal tubes (fat arrows) appear to have formed on the rostral side. (F) Cross-section through a 9-week regenerate in the growth beyond stage that had not recovered function. Axon regeneration appears to be sloppy in that axons are wisping dorsally towards the tissue wound that was created to gain access to the spinal cord. R, rostral; C, caudal; D, dorsal; V, ventral. Scale bars: 500 μm ((A-E) are the same scale); 200 μm (F). [file 1749-8104-6-1-S8.PDF]

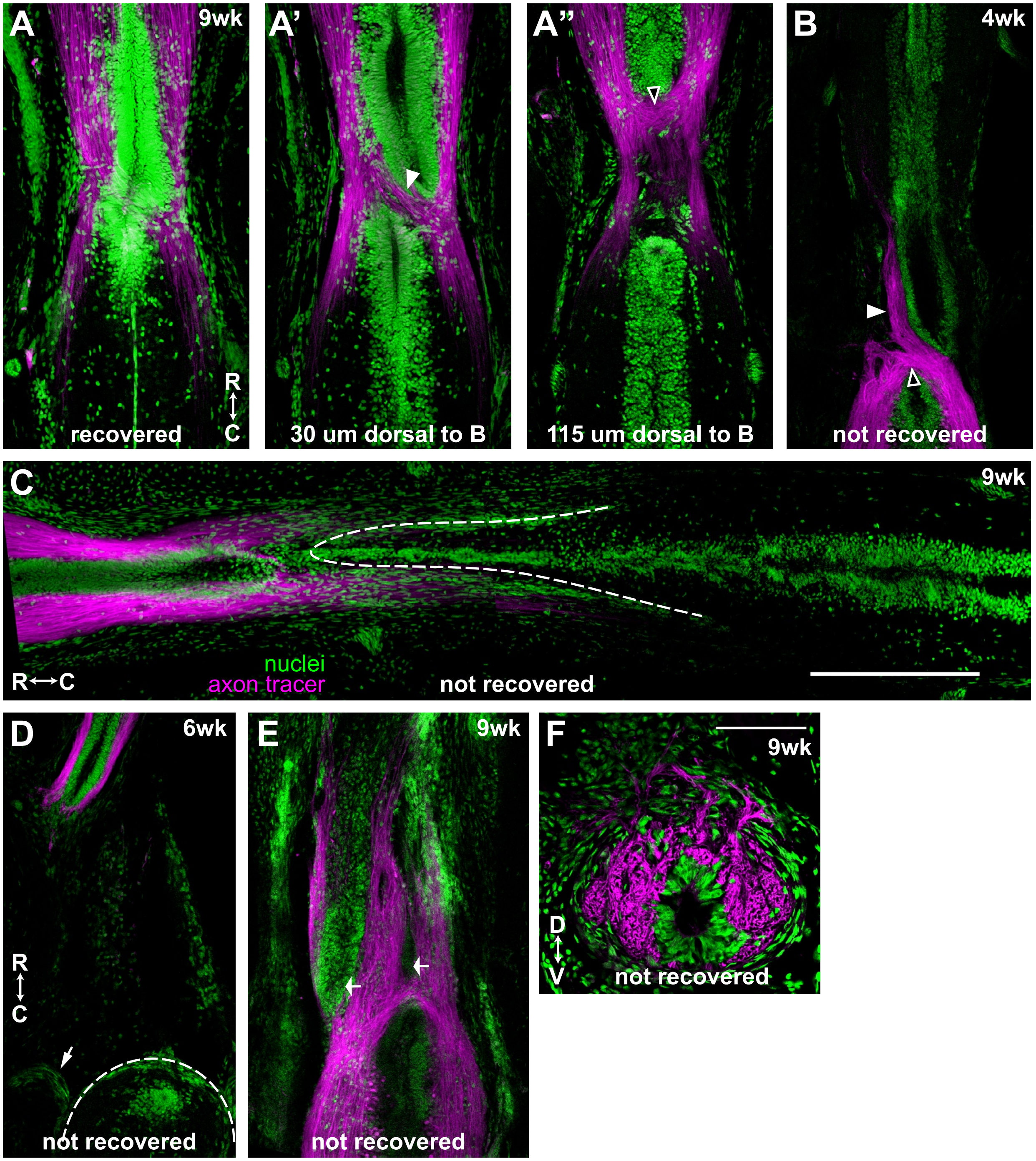

Additional file 8: Figure S4. Sloppiness and misalignments in axon regeneration. Axon regeneration has some tolerance for sloppiness, but gross misalignments may result in a delay or failure to recover. All images are montages of single confocal planes of longitudinal thick sections, except (F) which is a cross-section. Axons in all images were labeled with the axon tracer (magenta), and nuclei are green. **(A-A")** A 9-week regenerate in the growth beyond stage that had recovered swimming function. Gross misalignments are not present. The ependymal tubes have connected to re-establish a continuous central canal (A), yet axon regeneration appears to be a little sloppy in that some axons appear to decussate (arrowhead in (A')) and wrapping axons are still evident (open arrowhead in (A")). This, however, has not interfered with functional recovery. **(B)** A 4-week regenerate in the growth beyond stage that had not recovered function. Similar to (A), axons appear to wrap (open arrowhead) and decussate (arrowhead). This animal may not have recovered yet because it was sacrificed at such an early time point. **(C)** A 9-week regenerate in the growth beyond stage that had not recovered function. A long spike appears to have elongated from the caudal side (dotted lines), and this may indicate that the gap in the cord was large. Recovery, therefore, may be delayed. **(D)** A 6-week regenerate in the spiking stage that had not recovered function. The whole spike appears to be veering off in the wrong direction and does not line up properly with the cord on the other side (dotted lines). This gross misalignment may have prevented this animal from ever recovering function. Note that the whole spike, made up of meningeal cells, axons, and EG, is moving as unit in the wrong direction. Arrow, dorsal root to the spinal ganglia. **(E)** A 9-week regenerate in the growth beyond stage that had not recovered function. Two ependymal tubes (fat arrows) appear to have formed on the rostral side. The more prominent one is misaligned, and the less prominent one is properly aligned. Thus, the less prominent one may be forming to correct the misalignment. Recovery may be delayed as a result. **(F)** Cross-section through a 9-week regenerate in the growth beyond stage that had not recovered function. Axon regeneration appears to be sloppy in that axons are wisping dorsally towards the tissue wound that was created to gain access to the spinal cord. Recovery may be delayed as a result. R, rostral; C, caudal; D, dorsal; V, ventral. Scale bars: 500 μm ((A-E) are the same scale); 200 μm (F).
