# Supplementary material for: Meningeal cells and glia establish a permissive environment for axon regeneration after spinal cord injury in newts
Source: Neural Dev. 2011 Jan 4;6:1. doi: 10.1186/1749-8104-6-1 (PMC3025934; doi:10.1186/1749-8104-6-1)
Supplement: Additional file 11 — Table S1: Stages of 2.5- to 3-week regenerates analyzed after initial study. [file 1749-8104-6-1-S11.PDF]

Additional file 11: Table S1. Stages of 2.5 to 3 wk regenerates analyzed after initial study.

Exp-Animal	Prep	Antibody/ Stain	Axon Label	Stage on Rostral Side*	Stage on Caudal Side*
3wk-thick-1-1	thick	FN	rostral BDA app	spiking w/et (wide)	wisping (est)
3wk-thick-1-2	thick	FN	rostral BDA app	contact	contact
3wk-thick-1-3	thick	ColXII	rostral BDA app	growth beyond	growth beyond
3wk-thick-1-4	thick	ColXII	rostral BDA app	spiking w/et (wide)	wisping (est)
3wk-thick-1-5	thick	GFAP/GS	rostral BDA app	growth initiation/ wrapping	wisping (est)
3wk-thick-1-6	thick	GFAP/GS	rostral BDA app	growth beyond	growth beyond
3wk-thick-1-7	thick	GFAP/GS	rostral BDA app	spiking w/et (wide)	wisping (est)
3wk-thick-2-1	thick	TN	3A10	spiking w/et	wisping
3wk-thick-2-2	thick	LM	3A10	contact/ growth beyond	contact/ growth beyond
3wk-thick-2-3	thick	LM	3A10	wrapping/wisping	wisping
3wk-thick-2-4	thick	CSPG	3A10	wisping	wisping
3wk-thick-2-5	thick	TN	3A10	wisping/spiking w/o et	spiking w/o et
3wk-thick-2-6	thick	CSPG	3A10	spiking w/et (wide)	wisping
3wk-thick-2-8	thick	CSPG	3A10	contact	contact
cellID1-3wk-1	pfn long	LM, TN-C, GFAP	rostral BDA app 3A10	wrapping	–
cellID1-3wk-2	pfn long	CD11b	caudal BDA app 3A10	wisping	wisping
cellID1-3wk-3	pfn long	IB4, GFAP, LM, TN-C	rostral BDA app 3A10	wisping	wisping
cellID1-3wk-4	pfn long	FN, ColXII, CSPG	caudal BDA app 3A10	wisping	wisping
cellID1-3wk-5	pfn long	FN, CSPG, ColXII, IB4	rostral BDA app 3A10	wisping/spiking w/o et	wisping
cellID1-3wk-6	pfn long	FN, CSPG, ColXII, TN	caudal BDA app 3A10	wrapping/wisping	wisping
cellID1-3wk-7	pfn long	LM, TN, GFAP, IB4	rostral BDA app 3A10	wisping	wisping
cellID1-3wk-8	pfn long	LM, TN, GFAP, IB4	caudal BDA app 3A10	contact	contact
cellID4-3wk-9	cryo long	various	3A10	wisping	spiking w/o et
cellID4-3wk-10	cryo long	various	3A10	wrapping/wisping	wisping
cellID4-3wk-11	cryo long	various	3A10	wrapping/wisping	wisping
cellID4-3wk-12	cryo long	various	3A10	wrapping/wisping	wrapping
cellID6-3wk-13	pfn long	H&E	-	contact (est)	contact (est)
cellID6-3wk-14	pfn long	H&E	-	wisping (est)	wisping (est)
cellID8-3wk-1	pfn long	H&E, GFAP/GS	-	contact (est)	contact (est)
cellID8-3wk-2	pfn long	H&E, fibrin, GFAP/GS	-	wisping (est)	wisping (est)

Additional file 11: Table S1. (continued).

Exp-Animal	Prep	Antibody/ Stain	Axon Label	Stage on Rostral Side*	Stage on Caudal Side*
cellID8-3wk-3	pfn long	H&E, fibrin, GFAP/GS	-	contact (est)	contact (est)
2.5 wk-1-1	pfn long	vWF/FN, GFAP/fibrin	rostral BDA app 3A10	wisping	wisping
2.5 wk-1-2	pfn long	vWF/FN, GFAP/fibrin	rostral BDA app 3A10	wisping	wisping
2.5 wk-1-3	pfn long	vWF/FN, GFAP/fibrin	rostral BDA app 3A10	wisping	wrapping
2.5 wk-1-4	pfn long	vWF/FN, GFAP/fibrin	3A10	wisping	wisping
2.5 wk-1-5	cryo long	CSPG	3A10	wisping	wisping
2.5 wk-1-6	cryo long	CSPG	3A10	wrapping/wisping	wisping
2.5 wk-1-7	cryo long	CSPG	3A10	wisping	wisping
2.5 wk-1-8	cryo long	CSPG	3A10	wisping	wisping
2.5 wk-1-9	cryo cross	CSPG vWF/FN	3A10	wisping	wisping
2.5 wk-1-10	cryo cross	CSPG vWF/FN	3A10	wisping	wisping
2.5 wk-2-11	pfn long	H&E, GFAP, vWF	3A10	wrapping/wisping	wrapping/wisping
2.5 wk-2-12	pfn long	H&E, GFAP, vWF	3A10	wisping	wisping
2.5 wk-2-13	pfn long	H&E, GFAP, vWF	3A10	wisping	wisping
2.5 wk-2-14	pfn cross	GFAP, LM, TN	3A10	wisping (est)	wisping (est)
2.5 wk-2-15	pfn cross	GFAP, LM, TN	3A10	wrapping/wisping (est)	wrapping/wisping (est)
2.5 wk-2-16	pfn cross	GFAP, LM, TN	3A10	wrapping/wisping (est)	wisping (est)
2.5 wk-2-17	pfn cross	FN, ColXII, vWF	rostral BDA app	wisping (est)	wisping (est)
2.5 wk-2-18	pfn cross	FN, ColXII, vWF	rostral BDA app	spiking w/et (est)	wrapping (est)
2.5 wk-2-19	pfn cross	FN, ColXII, vWF	rostral BDA app	wrapping (est)	wisping (est)

* Stage was estimated (*est*) if axons were not labeled with BDA or 3A10 or if the tissue was analyzed in cross sections (*cross*). Staging in thick sections (whole-mount) is more reliable than staging in paraffin (*pfn*) or cryosections (*cryo*) sections. *long*, longitudinal section; *et*, ependymal tube.
