# Supplementary material for: Meningeal cells and glia establish a permissive environment for axon regeneration after spinal cord injury in newts
Source: Neural Dev. 2011 Jan 4;6:1. doi: 10.1186/1749-8104-6-1 (PMC3025934; doi:10.1186/1749-8104-6-1)
Supplement: Additional file 12 — Figure S5: a glia limitans-like structure may be present during the wrapping stage. Longitudinal sections through wrapping stage regenerates. Axons were labeled with the axon tracer in (C, D) and are shown in magenta. Each ECM protein is shown in green, and nuclei are blue. (A-D) Col XII (A), FN (B), and TN-C (C) expression wraps around the end of the cord, while LM (D) expression does not. R, rostral; C, caudal. Scale bar: 200 μm (A-D). [file 1749-8104-6-1-S12.PDF]

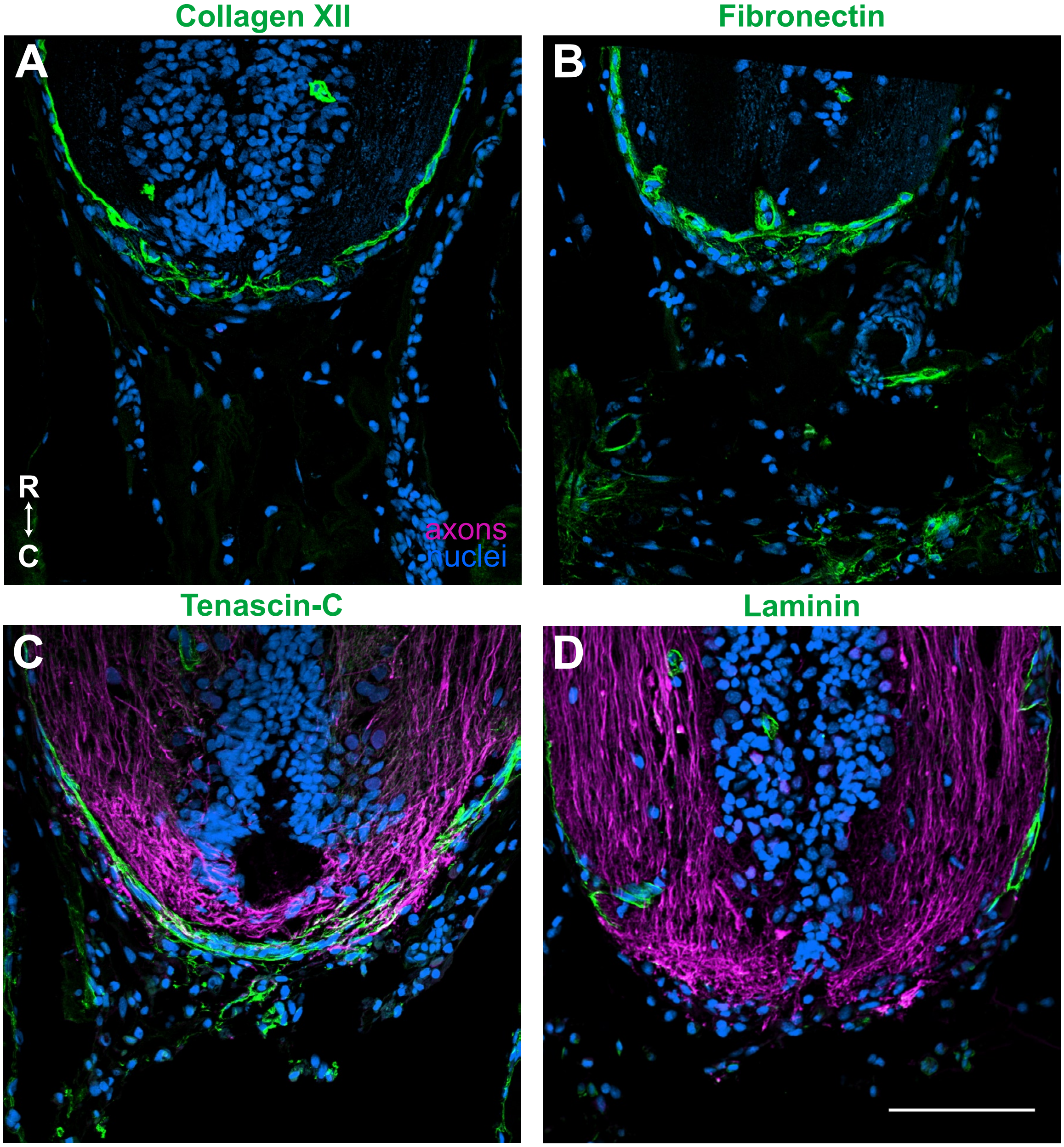

Additional file 12: Figure S5. A glia limitans-like structure may be present during the wrapping stage. Longitudinal sections through wrapping stage regenerates. Axons were labeled with the axon tracer in (C,D) and are shown in magenta. Each ECM protein is shown in green, and nuclei are blue. **(A-D)** Col XII (A), FN (B), and TN-C (C) expression wraps around the end of the cord, while LM (D) expression does not. R, rostral; C, caudal. Scale bar: 200 µm (A-D).
