# Supplementary material for: Meningeal cells and glia establish a permissive environment for axon regeneration after spinal cord injury in newts
Source: Neural Dev. 2011 Jan 4;6:1. doi: 10.1186/1749-8104-6-1 (PMC3025934; doi:10.1186/1749-8104-6-1)
Supplement: Additional file 13 — Table S2: Table of other antibodies tested. [file 1749-8104-6-1-S13.PDF]

Additional file 13: Table S2. Table of other antibodies tested.

Antigen	Antibody Type	Company, Cat #	Frozen	Paraffin	Thick	Dilution, Format	Notes
Collagen I (human)	goat IgG pAb	Santa Cruz, SC-25974	✖	-	-	1/10, concentrate	non-specific labeling
NCAM (chick)	mouse IgG1 mAb	DSHB, 4D	✖	-	-	1/25, 1/5, concentrate	no bands on reduced & non-reduced WBs
NCAM	mouse IgG1	DSHB, 5B8	-	-	-	ascites	no bands on reduced & non-reduced WBs
Glutamine Synthetase (sheep)	mouse IgG2a	BDTransLab, 610517	✔	✔	✔	~1/1500, concentrate	labels astros & all processes (astros & EG)
Keratin 18 (mammalian)	mouse IgG1 mAb	Santa Cruz, SC-32329	✔	✔	-	1/25, concentrate	labels EG in intact cord, but not regen cord
vimentin	mouse IgM	DSHB, 3CB2	✖	✖	-	1/50, concentrate	tested on intact cord
Sox2 (human)	rabbit IgG pAb	Abcam, ab15830	✔	-	-	1/>25, concentrate	tested on 3wk regen, appears to label neurons
msx 1/2 (chick)	mouse IgG1	DSHB, 4G1	✖ (in SCI)	-	-	1/100, ascites	tested on 3wk regen, no labeling in SCI
22/18	mouse IgM	DSHB, 22/18	✖ (in SCI)	-	-	1/25, concentrate	tested on 3wk regen, no labeling in SCI
Isolectin B4-FITC	-	Sigma, L2895	✔	✔	✔	use at 20 μg/ml	labels microglia, meninges, and many cells in lesion – not specific
Iba1	rabbit	Wako, 019-19741	✖	✖	-	1/500	tested on 3wk regen
Iba1 (human)	goat IgG pAb	Abcam, ab5076	✖	-	-	1/200, concentrate	tested on 3wk regen
OX-42	mouse	Serotec, Mca275r	-	✖	-	1/50	tested on 3wk regen
CD11b	rat	Serotec, Mca74ga	✖	✖	-	1/100	tested on 3wk regen
CD11b	rat	BD Pharm, 550282	✖	✖	-	1/50	tested on 3wk regen
F4/80	rat	Serotec, Mca497	-	✖	-	1/10	tested on 3wk regen
NG2	rat	US Biologicals, C5067-70d	✖	✖	-	1/500	tested on 3wk regen
NG2 (human)	goat IgG pAb	Santa Cruz, SC-30923 (G-20)	✖	-	-	1/25, concentrate	tested on 3wk regen
Raldh2 (human)	goat IgG pAb	Santa Cruz, SC-22592 (N-20)	✖	-	-	1/25, concentrate	tested on 3wk regen
Raldh2	rabbit pAb	from P. McCaffery via S. Scott	✖	✖	-	1/100	tested on intact cord
Olig1 (human)	goat IgG pAb	R&D Systems, AF2417	✖	-	-	1/25, concentrate	tested on 3wk regen
Olig2	mouse IgG1	Abcam, ab64547	-	✖	-	1/2000, ascites	tested on 3wk regen
olig	mouse IgG	DSHB, Olig	✖	✖	-	1/25, concentrate	tested on intact cord
MAG (chick)	mouse IgG1 mAb	Chemicon, MAB1567 (513)	✔	✖	✖	1/100, concentrate	
MAG	mouse mAb	from R. Quarles via M. Filbin	✖	-	-	1/50	tested on intact cord
Nogo-A (human)	rabbit pAb	Santa Cruz, SC-25660 (H-300)	✖	-	-	1/25, 1/10, concentrate	
P0	rabbit pAb	from M. Filbin	✔	-	-	1/50	tested on intact cord, strong label in PNS, weak label in CNS that may be non-specific, multiple bands on WB
