# Supplementary material for: Meningeal cells and glia establish a permissive environment for axon regeneration after spinal cord injury in newts
Source: Neural Dev. 2011 Jan 4;6:1. doi: 10.1186/1749-8104-6-1 (PMC3025934; doi:10.1186/1749-8104-6-1)
Supplement: Additional file 14 — Figure S6: meninges of the intact spinal cord. Longitudinal section through the intact spinal cord imaged with EM. (A) Region containing the meninges. p, pia mater; a, arachnoid mater; d, dura mater; sas, subarachnoid space; ecm, ECM; ef, glial end feet; o, oligodendrocyte. (B) Enlargement of box B in (A) showing skinny, dark processes of dura mater cells (arrows) associated with collagen fibrils (c) of the dura mater. (C) Enlargement of box C in (A) showing layers of arachnoid cell processes (a), a skinny process from a dura mater cell (arrow), and collagen (c). (D) Enlargement of box D in (A) showing a pia mater cell. (E) Enlargement of box E in (A) showing neural collagen (nc). (F) Enlargement of box F in (A) showing the basement membrane (arrowhead) at the glia limitans of the spinal cord, collagen (c) and neural collagen (nc). R, rostral; C, caudal. Scale bars: 15 μm (A); 3 μm (D); 1.5 μm (B, C, E, F). [file 1749-8104-6-1-S14.PDF]

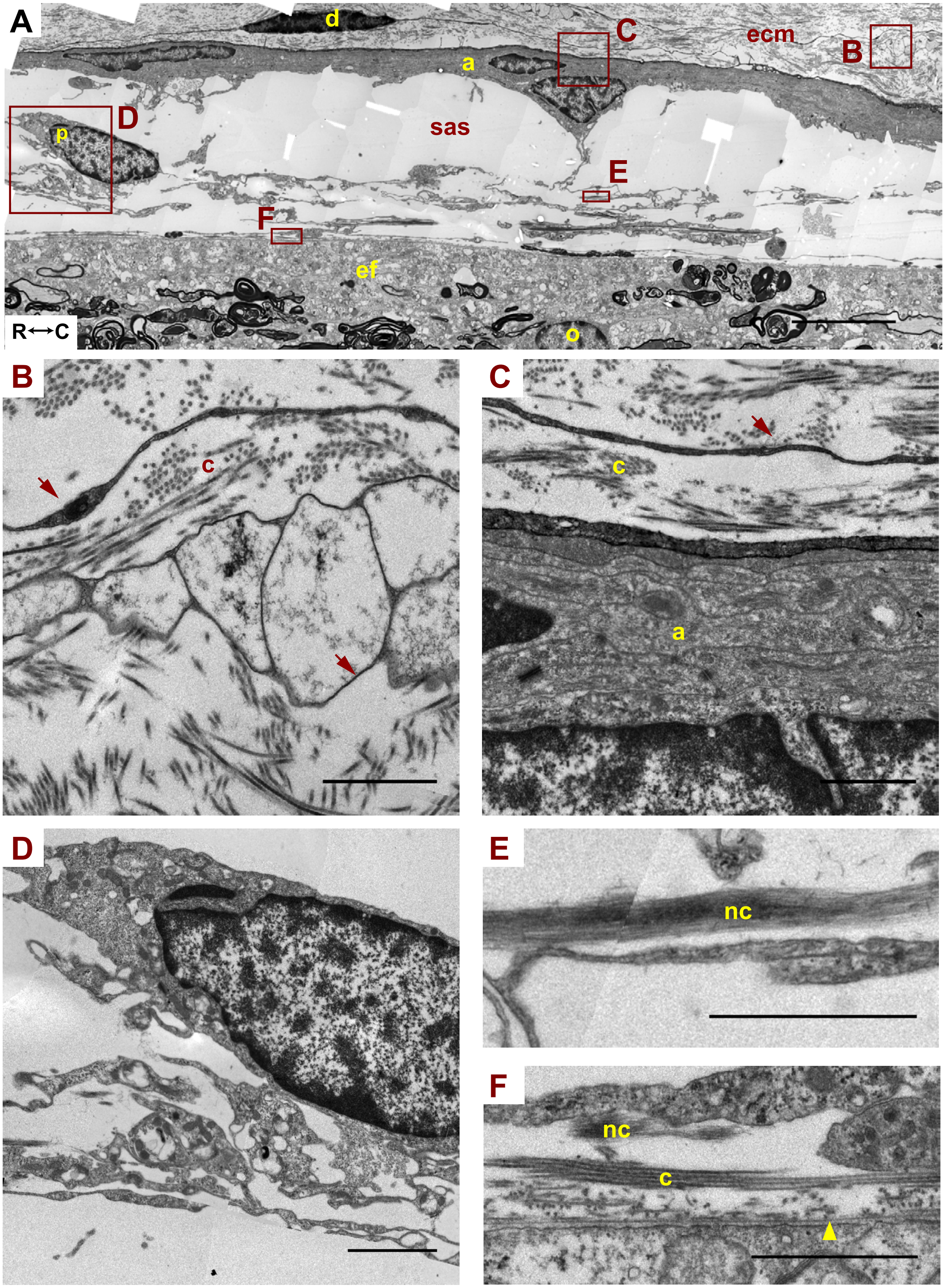

Additional file 14: Figure S6. Meninges of the intact spinal cord. Longitudinal section through the intact spinal cord imaged with EM. **(A)** Region containing the meninges. p, pia mater; a, arachnoid mater; d, dura mater; sas, subarachnoid space; ecm, ECM; ef, glial end feet; o, oligodendrocyte. **(B)** Enlargement of box B in (A) showing skinny, dark processes of dura mater cells (arrows) associated with collagen fibrils (c) of the dura mater. **(C)** Enlargement of box C in (A) showing layers of arachnoid cell processes (a), a skinny process from a dura mater cell (arrow), and collagen (c). **(D)** Enlargement of box D in (A) showing a pia mater cell. **(E)** Enlargement of box E in (A) showing neural collagen (nc). **(F)** Enlargement of box F in (A) showing the basement membrane (arrowhead) at the glia limitans of the spinal cord, collagen (c) and neural collagen (nc). R, rostral; C, caudal. Scale bars: 15 µm (A); 3 µm (D); 1.5 µm (B,C,E,F).
