# Supplementary material for: Meningeal cells and glia establish a permissive environment for axon regeneration after spinal cord injury in newts
Source: Neural Dev. 2011 Jan 4;6:1. doi: 10.1186/1749-8104-6-1 (PMC3025934; doi:10.1186/1749-8104-6-1)
Supplement: Additional file 15 — Figure S7: other cell types in the intact spinal cord. Longitudinal section through the intact spinal cord imaged with EM. (A) Region containing the central canal (cc), EG layer (EG) and a portion of the grey mater (gm). (B) Enlargement of box B in (A) showing astrocytes. (C) Enlargement of box C in (A) showing the cytoplasm of light EG. (D) Enlargement of box D in (A) showing the cytoplasm of dark EG. (E) A microglial cell. (F) An oligodendrocyte. R, rostral; C, caudal. Scale bars: 15 μm (A, B, F); 5 μm (E); 3 μm (D); 1.5 μm (C). [file 1749-8104-6-1-S15.PDF]

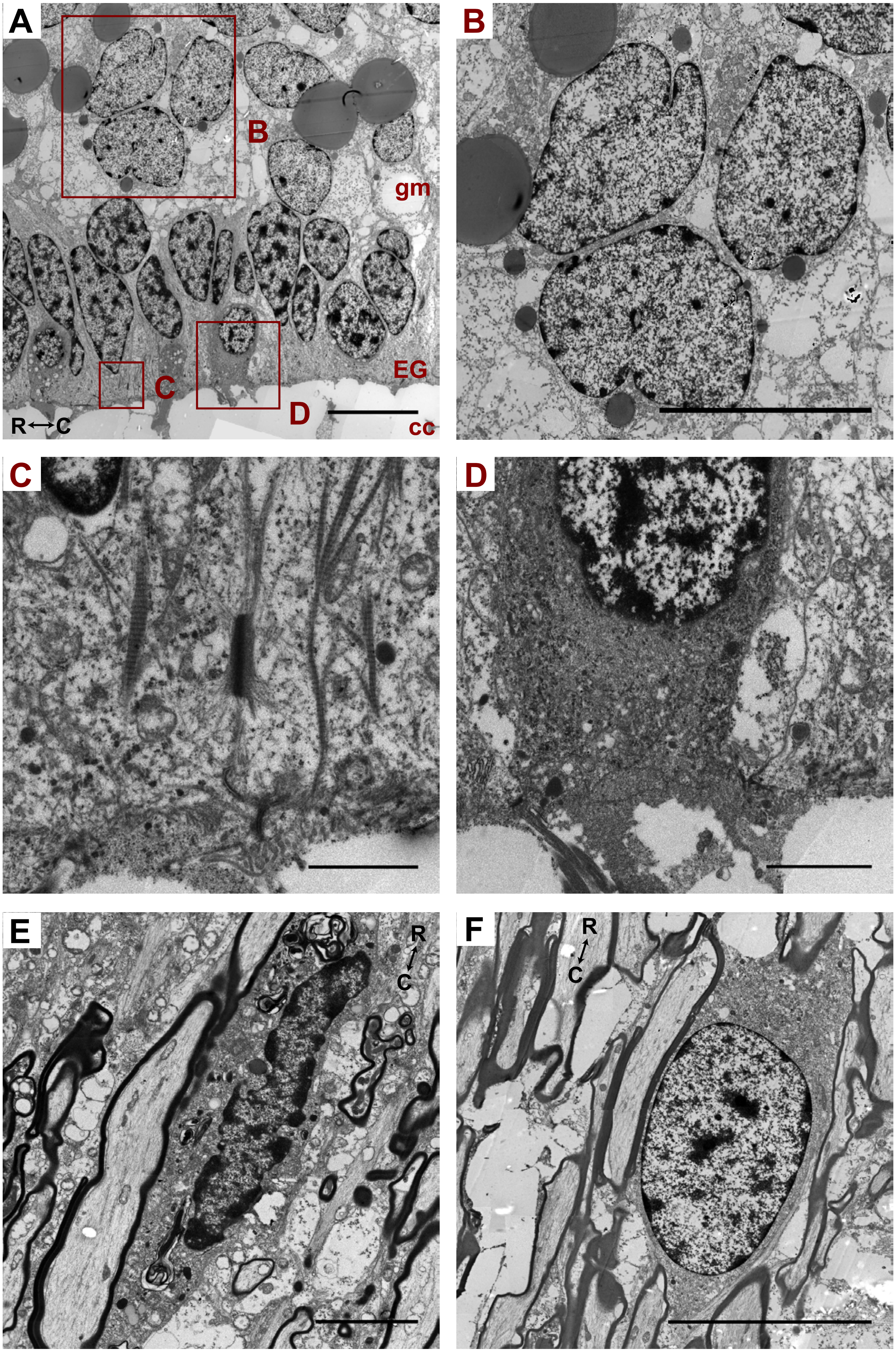

Additional file 15: Figure S7. Other cell types in the intact spinal cord. Longitudinal section through the intact spinal cord imaged with EM. **(A)** Region containing the central canal (cc), EG layer (EG) and a portion of the grey mater (gm). **(B)** Enlargement of box B in (A) showing astrocytes. **(C)** Enlargement of box C in (A) showing the cytoplasm of light EG. **(D)** Enlargement of box D in (A) showing the cytoplasm of dark EG. **(E)** A microglial cell. **(F)** An oligodendrocyte. R, rostral; C, caudal. Scale bars: 15 µm (A,B,F); 5 µm (E); 3 µm (D); 1.5 µm (C).
