# Supplementary material for: Meningeal cells and glia establish a permissive environment for axon regeneration after spinal cord injury in newts
Source: Neural Dev. 2011 Jan 4;6:1. doi: 10.1186/1749-8104-6-1 (PMC3025934; doi:10.1186/1749-8104-6-1)
Supplement: Additional file 16 — Figure S8: glia of the regenerating spinal cord. (A-D) Longitudinal section through a wisping stage regenerate imaged with EM. (A) Region containing axons wisping ahead of the TV. (B) Enlargement of box B in (A) showing a light glial process (EGL) that is associated with regenerating axons (ax). (C) Enlargement of box C in (A) showing light (EGL) and dark (EGD) EG lining the TV. (D) Enlargement of box D in (A) showing a dark EG process (EGD) associated with axons (ax). R, rostral; C, caudal. Scale bars: 50 μm (A); 15 μm (C); 5 μm (B); 3 μm (D). [file 1749-8104-6-1-S16.PDF]

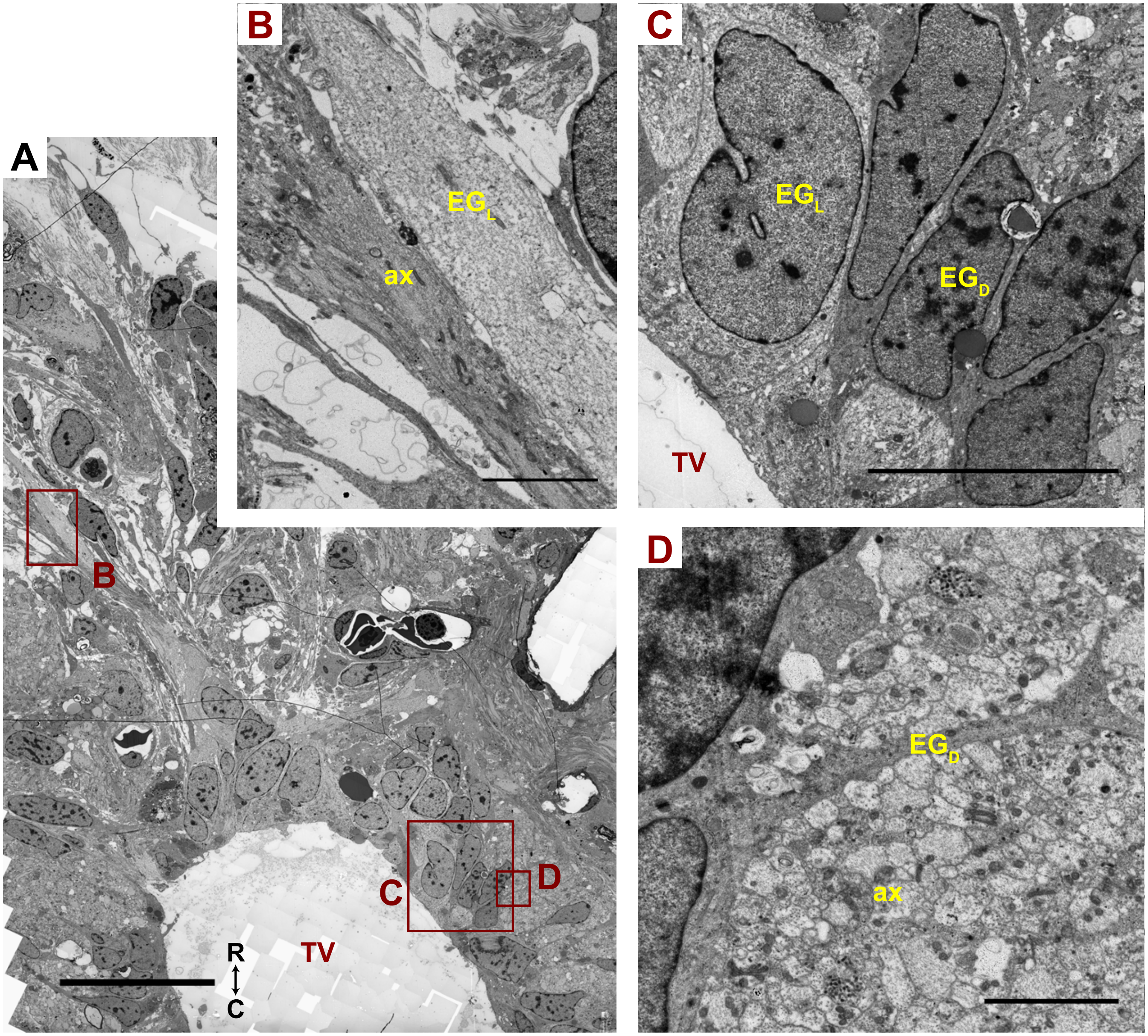

Additional file 16: Figure S8. Glia of the regenerating spinal cord. (**A-D**) Longitudinal section through a wisping stage regenerate imaged with EM. **(A)** Region containing axons wisping ahead of the TV. **(B)** Enlargement of box B in (A) showing a light glial process (EG_L) that is associated with regenerating axons (ax). **(C)** Enlargement of box C in (A) showing light (EG_L) and dark (EG_D) EG lining the TV. **(D)** Enlargement of box D in (A) showing a dark EG process (EG_D) associated with axons (ax). R, rostral; C, caudal. Scale bars: 50 µm (A); 15 µm (C); 5 µm (B); 3 µm (D).
