# Supplementary material for: Meningeal cells and glia establish a permissive environment for axon regeneration after spinal cord injury in newts
Source: Neural Dev. 2011 Jan 4;6:1. doi: 10.1186/1749-8104-6-1 (PMC3025934; doi:10.1186/1749-8104-6-1)
Supplement: Additional file 17 — Figure S9: the inflammatory response in early and late stage regenerates. (A,B) Adjacent longitudinal sections through a 1-week regenerate labeled with an anti-fibrin antibody (A) and stained with H&E (B). A fibrin clot is formed in the injury site (A), and inflammatory cells can already be identified in this clot (B). (B',B") Enlargement of primed and double primed boxes in (B). Lymphocytes (arrowheads in (B')) and monocytes (arrow in (B")) can be identified. Asterisk, fibrin clot. (C) Longitudinal section through a contact stage regenerate. A relatively strong inflammatory response has not prevented this animal from progressing to this late stage. (C') Enlargement of box in (C). Lymphocytes (arrowhead) and monocytes (arrows) can be identified. R, rostral; C, caudal. Scale bars: 200 μm (A; (B,C) are the same scale); 50 μm ((B',B") are the same scale; C'). [file 1749-8104-6-1-S17.PDF]

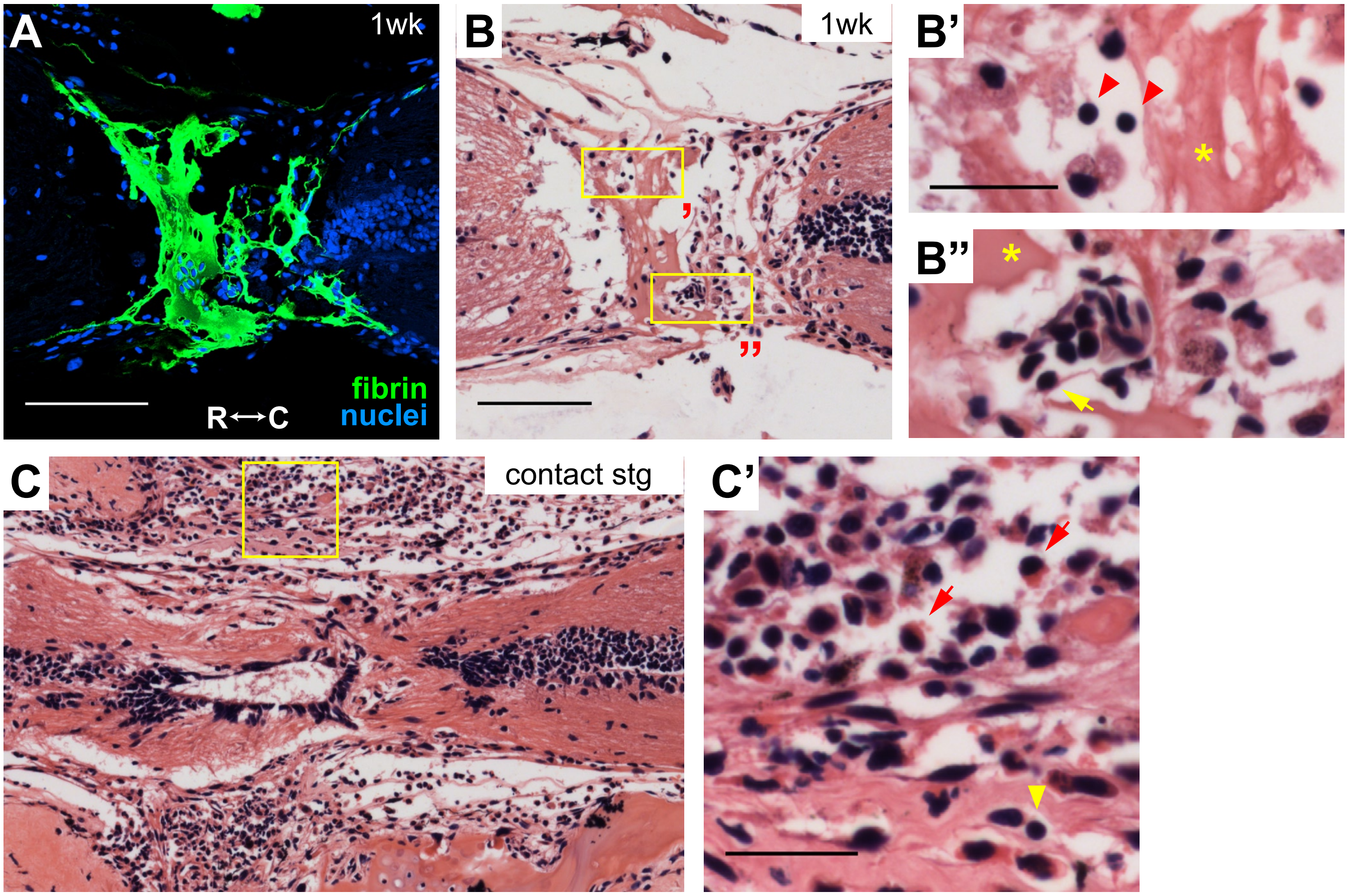

Additional file 17: Figure S9. The inflammatory response in early and late stage regenerates. **(A,B)** Adjacent longitudinal sections through a 1-week regenerate labeled with an anti-fibrin antibody (A) and stained with H&E (B). A fibrin clot is formed in the injury site (A), and inflammatory cells can already be identified in this clot (B). **(B',B")** Enlargement of primed and double primed boxes in (B). Lymphocytes (arrowheads in (B')) and monocytes (arrow in (B")) can be identified. Asterisk, fibrin clot. **(C)** Longitudinal section through a contact stage regenerate. A relatively strong inflammatory response has not prevented this animal from progressing to this late stage. **(C')** Enlargement of box in (C). Lymphocytes (arrowhead) and monocytes (arrows) can be identified. R, rostral; C, caudal. Scale bars: 200 µm (A; (B,C) are the same scale); 50 µm ((B',B") are the same scale; C').
